# Vaccine elicitation of HIV broadly neutralizing antibodies from genome-edited B cells in non-human primates and derived lymphoid organoids

**DOI:** 10.1038/s41434-026-00610-8

**Published:** 2026-04-10

**Authors:** Mary Tenuta, Morgan Bravo, Alex Olson, Celia L. Saney, Jason Weinfurter, Elana Ben-Akiva, Disha Bhange, Christopher A. Cottrell, Logan Vosler, Kimberly Weisgrau, Dennis R. Burton, Darrell J. Irvine, William R. Schief, Eva Rakasz, Chester J. Joyner, James E. Voss

**Affiliations:** 1 Department of Immunology and Microbiology, The Scripps Research Institute, La Jolla, CA USA; 2 Center for Vaccines and Immunology, College of Veterinary Medicine, University of Georgia, Athens, GA USA; 3 Center for Tropical and Emerging Global Diseases, University of Georgia, Athens, GA USA; 4 Department of Pathobiological Sciences, School of Veterinary Medicine, University of Wisconsin, Madison, WI USA; 5 Koch Institute for Integrative Cancer Research, Massachusetts Institute of Technology, Cambridge, MA USA; 6 Consortium for HIV/AIDS Vaccine Development (CHAVD), Scripps Research Institute, La Jolla, CA USA; 7 Immunology Services, Wisconsin National Primate Research Center, Madison, WI USA; 8 AIDS Vaccine Research Laboratory, University of Wisconsin, Madison, WI USA; 9 IAVI Neutralizing Antibody Center, The Scripps Research Institute, La Jolla, CA USA; 10 The Ragon Institute of Mass General, MIT and Harvard, Cambridge, MA USA; 11 Department of Biological Engineering, Massachusetts Institute of Technology, Cambridge, MA USA; 12 Department of Materials Science and Engineering, Massachusetts Institute of Technology, Cambridge, MA USA; 13 Howard Hughes Medical Institute, Chevy Chase, MD USA; 14 Moderna, Cambridge, MA USA; 15 Department of Infectious Diseases, College of Veterinary Medicine, University of Georgia, Athens, GA USA

**Keywords:** Translational immunology, Infectious diseases, Gene therapy

## Abstract

HIV broadly neutralizing antibodies (bnAbs) are promising reagents for prevention and therapy of disease; however, their elicitation is constrained by genetic limitations of the human B cell antigen-receptor (BCR) repertoire. Precision genome-editing offers a potential solution by enabling bnAb genes to be programmed into the BCR repertoire as *IgH*-modified B cells. Such cells can be vaccinated to elicit durable bnAb memory responses in mice; however, extending this success to non-human primates (NHPs) would be a major advance towards clinical translation. Here, we show that ex vivo reprogrammed NHP B cells can survive autologous infusion and respond to immunization, differentiating into antibody-secreting cells (ASCs) that can produce up to 1 µg/ml of a bnAb in serum following vaccination prime. Although durable transgenic memory responses were not generated, vaccination of engineered cells in secondary lymphoid organoid (SLO) cultures recapitulated transient ASC responses in vitro. These findings suggest that NHP-derived SLOs could provide a platform to optimize engineering and vaccination conditions that drive germinal center maturation of *IgH*-reprogrammed B cells in a clinically relevant NHP model, supporting the development of engineered B-cell vaccines that generate durable bnAb responses as a potential functional cure for HIV.

Small-molecule antiretroviral therapy (ART) has transformed HIV infection from a fatal disease into a manageable chronic condition for most of the 39 million people diagnosed with the virus [1]. By suppressing viral replication to undetectable levels, ART prevents transmission [2] and restores immune function [3, 4]. However, ART is not curative. A persistent reservoir of latently infected cells can reignite infection, necessitating lifelong treatment [5, 6]. This imposes substantial clinical and societal burdens, including emergence of drug-resistant virus following treatment interruption [7, 8], long-term toxicities [9], and high economic costs, particularly in resource-limited settings with infection rates as high as 23.4% [10, 11]. In the United States, HIV treatment accounts for an estimated $22.5–35.6 billion annually [12]. These challenges underscore the urgent need to achieve durable virologic control without continuous treatment [13].

Broadly neutralizing antibodies (bnAbs) have emerged as promising agents for HIV prevention and treatment due to their long serum half-lives and favorable safety profiles [14, 15]. Clinical studies show that bnAbs can prevent infection by sensitive strains [16], suppress viremia [17–22], and sustain viral control after ART discontinuation [23–30]. HIV bnAb treatment can augment host antiviral immunity [31–33] and lead to viral suppression even after antibody titers wane in some individuals [23, 24]. However, HIV bnAbs are exceptionally difficult to elicit by vaccination because they require features that can only be derived through the extensive affinity maturation of rare precursor B cell antigen receptors (BCRs) [34]. This limitation has prompted interest in gene therapy approaches to enable long-term bnAb production in vivo [35, 36].

One such approach circumvents repertoire-imposed limitations to bnAb elicitation by precisely inserting fully mature bnAb transgene cassettes into the immunoglobulin heavy chain (*IgH*) locus of primary B cells, enabling their expression as functional BCRs using cell endogenous regulatory elements and heavy chain (HC) constant genes [37–41]. In mice, *IgH*-reprogrammed B cells can be vaccinated to expand and undergo affinity maturation [42, 43], leading to the generation of long-lived plasma cells (LLPCs) that secrete isotype-switched bnAbs, as well as memory B cells that can be repeatedly boosted [44–46].

Elicitation of durable bnAb memory responses from *IgH*-reprogrammed B cells in non-human primates (NHPs) would represent a key step toward clinical translation and enable evaluation of such responses in SHIV-infected animals [47, 48]. Here, we describe the development of ex vivo genome-editing methods for Indian rhesus macaque B lymphocytes and demonstrate that *IgH*-reprogrammed cells can survive autologous infusion and respond to vaccination, generating robust, isotype-switched HIV bnAb serum titers associated with circulating antibody-secreting cells (ASCs). Responses were transient, unboostable, and lacked evidence of T cell-dependent germinal center (GC)-maturation, however, suggesting that engineered cells predominantly underwent extrafollicular maturation in response to immunization, and that alternative vaccines or engineering conditions might improve in vivo programming of GC-derived bnAb memory and LLPCs.

To address this problem, we established a secondary lymphoid organoid (SLO) culture system to model autologous engineered B cell responses to vaccination ex vivo. While *IgH*-reprogrammed cells failed to persist in unvaccinated organoids, immunized cultures supported their differentiation into ASCs that secreted transgenic antibody, recapitulating in vivo responses. These findings suggest that NHP SLOs may offer a physiologically relevant in vitro platform for optimizing engineered B cell vaccine parameters prior to in vivo NHP vaccination experiments.

## Results

### Activation and IgH reprogramming of NHP B cells

**Fig. 1:**
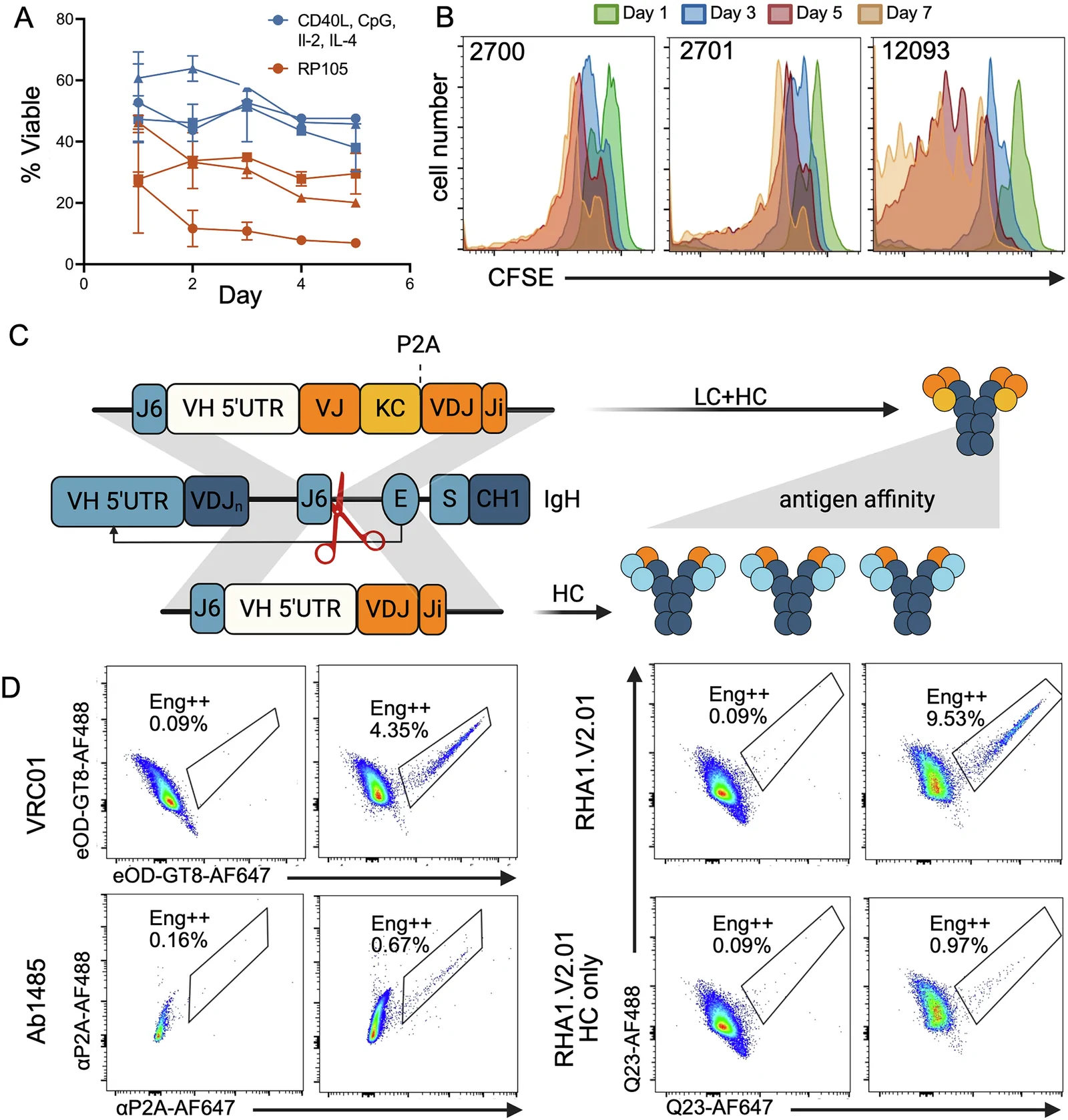
*IgH*-reprogramming of rhesus macaque primary B cells. **A** Viability of B cells cultured in pilot-1 activation media (CD40L, CpG, IL-2, IL-4) or RP105 was assessed over time by flow cytometry. **B** Carboxyfluorescein succinimidyl ester (CFSE) was used to track B cell division in pilot-1 media. Dilution of the CFSE stain intensity that occurs during rounds of cell division was monitored by flow cytometry on the indicated day of culture for B cells from three donor animals. **C** Reprogramming of the *IgH*-locus is achieved by insertion of an HIV bnAb donor cassette into a CRISPR–Cas9-induced DSB at the 3’ of the rhesus macaque *IgH-J6* gene. 500 bp HAs on either side of the cassette allow incorporation by HDR in dividing cells. The cassette encodes both the bnAb LC and HC variable regions (top), or only the HC variable region only (bottom). Expression is regulated by a 1.5 kb region from the 5’UTR of a rhesus macaque IGHV-gene, active only upon correct integration. The LC + HC cassette includes a leader, LC (species matched constant domain), P2A-peptide, HC variable region with leader, and a splice donor consensus sequence is preserved. P2A cleavage allows pairing of the LC and HC which is expressed using cell endogenous *IgH* constant genes. The HC-only design includes the 5′UTR, leader, HC variable region and splice donor. In HC-only cells, the new HC pairs with endogenous LCs, generating antibodies with variable antigen affinities. **D** The engineering strategies in (**C**) can be used to reprogram NHP B cells to express various human or authentic NHP bnAbs. Engineering efficiency can be assessed two or three days after engineering by flow cytometry using fluorophore conjugated probes that recognize the novel bnAb specificity.

To enable *IgH*-reprogramming, primary B cells must be activated to induce cell-cycle entry and enhance homology-directed DNA repair (HDR) at CRISPR-Cas9-induced double-strand breaks (DSBs) [49]. CD20+ B cells were purified from Indian rhesus macaque blood (Fig. S1A, B) and cultured with an anti-human RP105 (CD180) monoclonal antibody (mAb) reported to cross-react with NHP orthologues. This approach previously enabled engineering of human [38, 45] and mouse [38, 43] B cells, with murine cells demonstrating survival and function in vivo [43]. This mAb failed to activate NHP B cells, however (Fig. 1A), likely due to poor receptor binding (Fig. S1C). Optimal activation was subsequently achieved using recombinant human CD40L, IL-2, IL-4, and CpG, though mitogenic responses varied by donor (Fig. 1A, B).

Nucleofection conditions for optimal Cas9-ribonucleoprotein (RNP) delivery to activated B cells were identified (Fig. S2A), and ten guide RNAs (gRNAs) targeting the *IGHJ6* gene region were screened for their ability to generate DSBs (Fig. S2B, C). We based bnAb DNA homology-repair template (HRT) designs on an RNP that uses the conserved GG splice-donor site of *IGHJ6* as the Cas9 protospacer-adjacent motif (PAM). This generates a DSB at a site analogous to one targeted in our successful murine model (Fig. S2B) [44].

Our first NHP HRT was based on a VRC01 bnAb [50] design that resulted in reprogrammed B cells that could generate boostable memory responses after vaccination in mice [44]. This construct included ~500 base-pair (bp) homology arms (HAs) flanking a rhesus macaque IGHV-gene promoter segment, the VRC01 light chain (LC) variable region with rhesus kappa constant gene, and the VRC01 HC variable domain. The design preserves a splice donor sequence after the HC variable region to enable expression using cell endogenous *IgH* constant genes. A P2A self-cleaving peptide [51] positioned after the LC enables co-translational separation and pairing of VRC01 light and HCs (Fig. 1C). Engineering efficiencies were highest when cells were transduced with adeno-associated viral vector, serotype-6 (AAV6), carrying the HRT followed by nucleofection with RNP on day-3 of culture (Fig. S2D, E). *IgH*-reprogramming frequencies varied from 1 to 10% (Fig. S2F).

To test broader applicability, two bnAbs previously elicited in SHIV-infected NHPs were engineered into B cells: Ab1485 [52] and RHA1.V2.01 [53] directed to the V3-glycan and V2-apex epitopes of HIV Envelope (Env)-trimer, respectively. Both antibodies bind MD39-ferritin, the Env nanoparticle-immunogen which successfully elicited memory responses from VRC01-engineered B cells in mice [44] (Fig. S3A). These bnAbs also neutralized the autologous BG505-pseudovirus as recombinant IgG (Fig. S3B). We developed a second reprogramming strategy that inserts only the bnAb HC variable region into the *IgH*-locus (Fig. 1C). This design, suited for antibodies like RHA1.V2.01 that recognize antigen primarily using HC encoded paratopes, leverages endogenous LC pairing to generate pauci-clonal B cell populations with diverse affinities for the antigen [37]. It reduces HRT size, eliminates the need for a P2A-peptide, and restores expression of these chimeric BCRs from distinct HC and LC loci. Flow cytometry was used to evaluate cell surface expression for all transgenic BCRs. VRC01 and Ab1485 were detected using eOD-GT8 [54] antigen or an anti-P2A mAb. Cells engineered with RHA1.V2.01 HC showed ~10% binding to HIV Env Q23 [55] compared to LC + HC engineered populations (Fig. 1D).

### Characterization of IgH-reprogrammed cells for autologous infusion

**Fig. 2:**
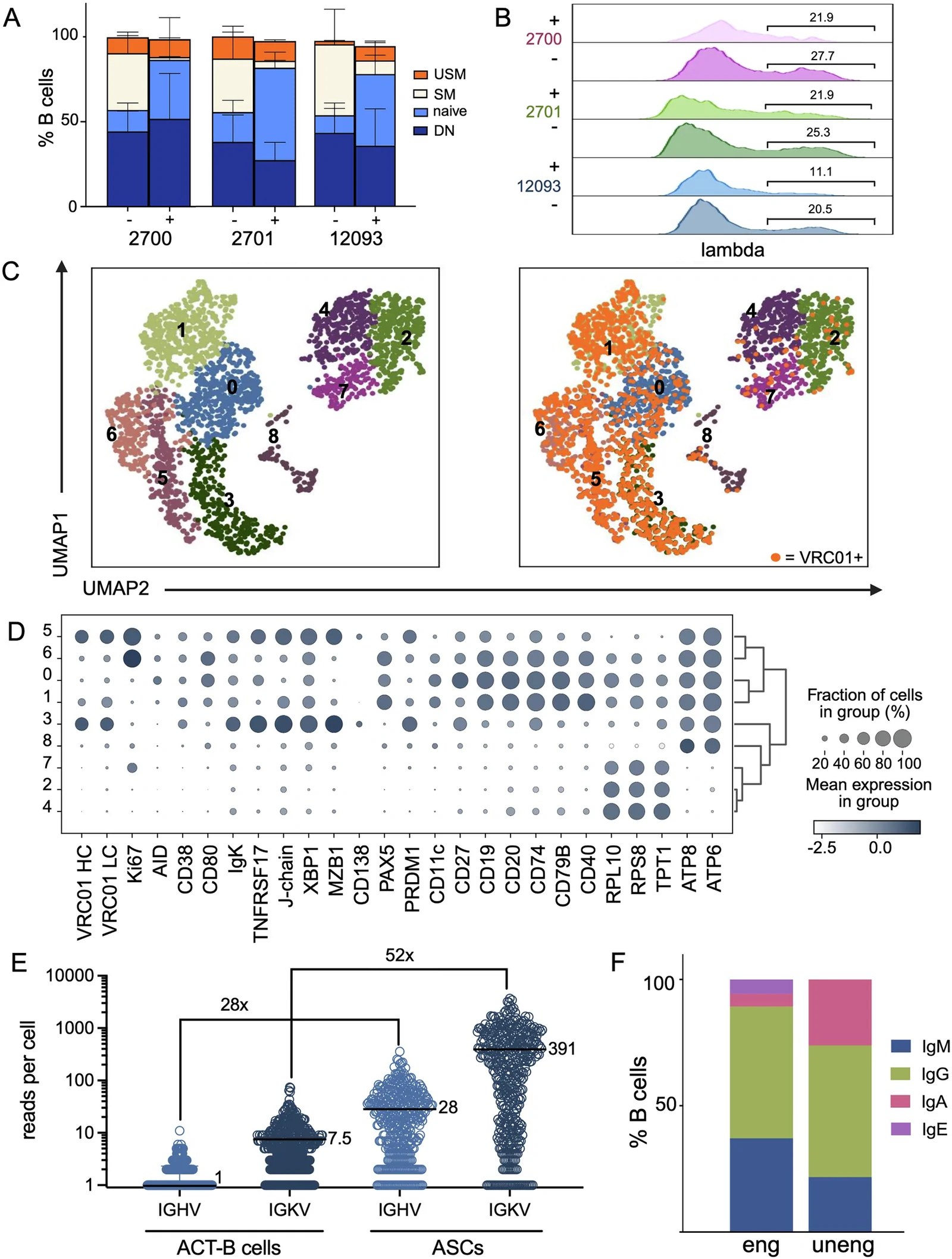
Characterization of VRC01 B cells for autologous engraftment and vaccination in NHPs. **A** The frequency of the various phenotypes of VRC01 expressing CD20 + B cells (+) gated on live, single cells that are KO11- and GT8 + +, or unengineered (-) B cells is shown. Phenotypes are defined as follows; USM (CD27 + , IgD + ), SM (CD27 + , IgD-), Naïve (CD27-, IgD+ ), and DN (CD27-, IgD-). **B** Histogram of VRC01 engineered (+) or unengineered (-) cells that are also cell surface lambda chain positive at the time of engraftment (day 6 of culture). **C** UMAP Leiden clusters from single cell RNA transcriptomics of an engineered pool of cells at the time of engraftment is shown above. Cells with VRC01 transcripts are indicated in orange (right). **D** Dotplots showing transcriptional levels of VRC01 and other specific genes of interest are shown across all clusters. Relatedness of each cluster is shown. **E** Number of sequencing reads per engineered cell that aligned to either VRC01 IGHV or IGKV genes on day-6 of culture in pilot-1 media, comparing cells belonging to “activated B cell” (ACT-B) clusters 0 and 1, or ASC clusters 3 and 5. Medians with interquartile ranges are indicated for each data set. Fold-increase of the IGHV or IGKV means between engineered activated B cells and ASCs are indicated. **F** Comparison of isotype frequencies of engineered cells compared to unengineered cells in ASC clusters 3 and 5 by single cell sequencing analysis.

We chose to initiate an NHP pilot study with VRC01-engineered cells and MD39-ferritin vaccinations as this combination previously elicited robust bnAb memory responses in mice [44]. VRC01-reprogrammed cells were therefore characterized by flow cytometry and single-cell RNA sequencing (scRNA-seq) 3-days post-reprogramming (Fig. S4A). Flow cytometry showed 80% of cells remained CD20⁺ and these could be subdivided into naïve (CD27-IgD+), unswitched memory (USM: CD2+⁺IgD+), switched memory (SM: CD27+IgD-), and double-negative (DN: CD27-IgD-) B cell subsets, as previously described [56] (Fig. 2A). DNs were further classified as DN1 (CD21+), DN2 (CD11c+), or DN3 (CD21-CD11c-), with DN3s dominating (Fig. S4B). DN3s appear to be DN2 precursors based on pseudotime trajectories [57], which are positioned on extrafollicular developmental pathways leading to short-lived ASCs in analogous human B cell populations [58–60]. To evaluate cell-surface co-expression of transgenic and native Ig in reprogrammed cells, we stained for endogenous lambda (λ) LCs. Only a slight reduction in λLC staining was seen in VRC01-edited cells relative to unengineered controls, indicating frequent co-expression (Fig. 2B). For scRNA-seq, engineered cells were fluorescence-activated cell sorting (FACS)-enriched and pooled with unmodified cells for 5’ barcoded cDNA library generation. A custom *Macaca mulatta* reference genome incorporating mature VRC01 V-genes and improved annotations was used to resolve eight populations based on the top 50 differentially expressed genes. Reprogrammed cells could be identified by detecting VRC01 V-gene transcripts. Engineered and unengineered cells overlapped in UMAP projections, indicating similar global transcriptional profiles (Fig. 2C). Population identities were assigned by gene transcription patterns (Fig. 2D). Large clusters 0 and 1 appeared to be activated B cells, with CD27 and CD80 levels distinguishing cluster 0 as more memory-like and cluster 1 as naïve. Interestingly, despite similar Ig-transcription levels (i.e. IGK), the naïve population appeared enriched in engineered cells (Fig. 2C, D, Fig. S4C). Clusters 3 and 5 are non-dividing and dividing ASCs, respectively, based on elevated Ki67 transcripts in the latter. Cluster 6 transcribes activated B cell genes and Ki67, indicating cells in cycle. Clusters 2, 4, 7, and 8 likely represent DNs identified by flow cytometry despite low apparent CD19/CD20 transcription levels. DNs had elevated mitochondrial, anti-apoptotic, and ribosomal gene transcription, consistent with extrafollicular ASC differentiation [61, 62]. VRC01-transcripts were 28-52-fold higher in ASC populations (clusters 3 and 5) relative to activated B cells (clusters 0 and 1) suggesting normal regulation of the transgenic *IgH*-locus. The VRC01 LC V-gene was 7.5-14x more readily detected compared with the HC even though the genes are derived from the same transcript, suggesting a potential DNA fragmentation bias during library preparation (Fig. 2E). Most clusters had low *IgH*-constant gene reads, often with multiple isotypes detected per cell likely due to transcription from constant-gene switch regions or residual cDNA from recent class-switching events. In plasma cell (PC) cluster 3 and plasmablast (PB) cluster 5, where *IgH*-transcription is significantly upregulated, isotype could be determined and revealed substantial class-switching to IgG and IgA, with VRC01+ cells significantly depleted for IgA (Fig. 2F, Fig. S4C). A second donor showed similar results (Fig. S5).

### Vaccinating NHPs after autologous infusion of IgH-reprogrammed cells induces high but transient VRC01 serum titers

**Fig. 3:**
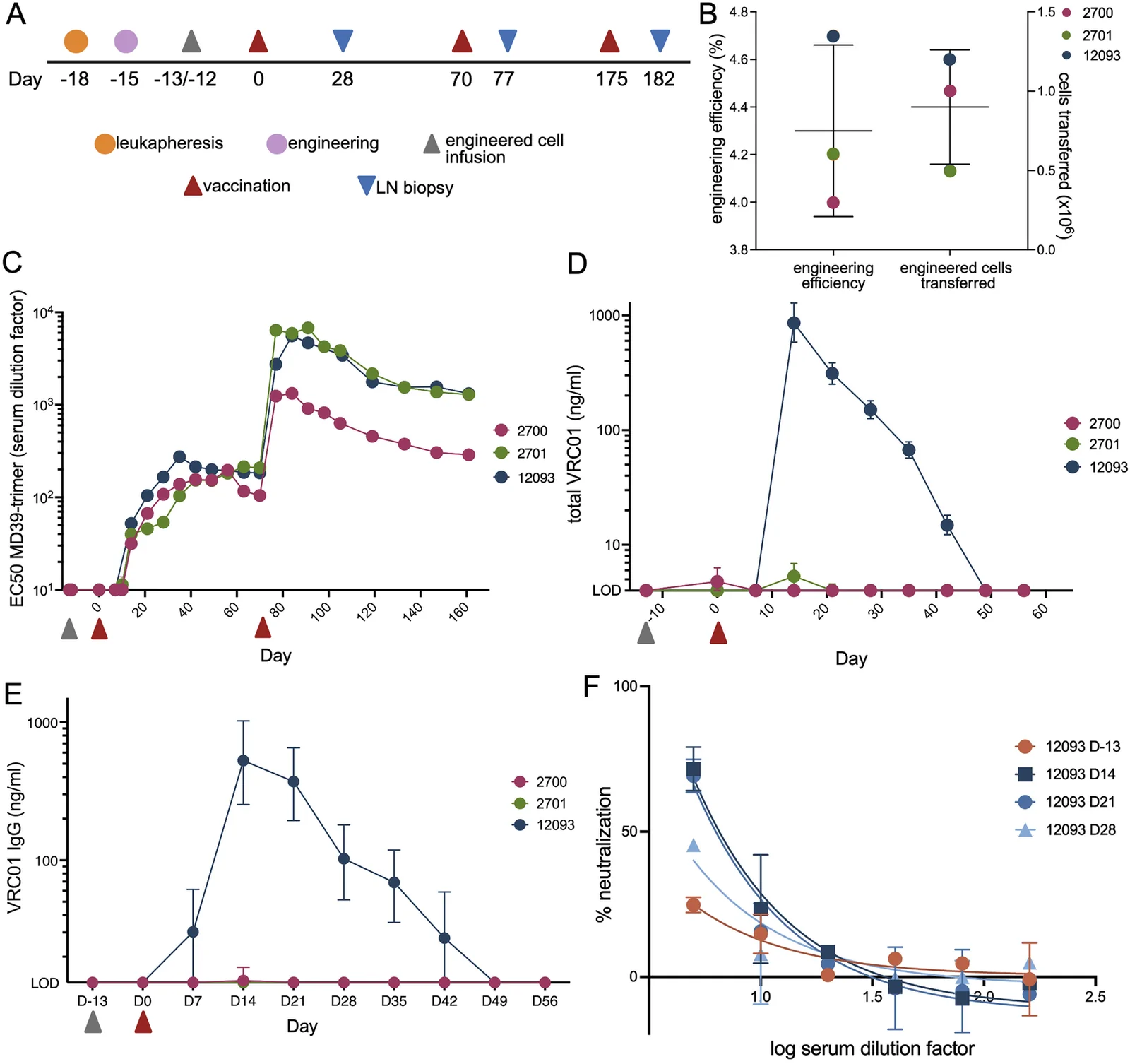
Serum responses show that VRC01 antibody can be elicited from *IgH*-reprogrammed cells by MD39-ferritin vaccination in NHPs. **A** Timeline for reprogrammed B cell transfer, vaccination, and sampling in three NHPs is shown. Blood draws were performed weekly for serum, PBMC, and plasma collection. **B** B cells from three leukapheresed animals were purified and activated in culture. They were targeted for *IgH*-reprogramming three days later and autologously infused back into donor animals on day 5 or 6. The *IgH*-reprogramming efficiencies were assessed by flow cytometry (left Y-axis) and total number of VRC01 B cells transferred back into each animal in our study is given (right Y-axis). **C** Total antigen-specific IgG-titers against the MD39-native trimer after homologous prime and boost using MD39-ferritin was assessed by ELISA and are reported as SDF EC50s. The gray triangle indicates when autologous B cells were intravenously infused into animals and the red triangles indicate immunization time points. **D** An anti-P2A ELISA was used to quantify total VRC01 antibody in animal sera at indicated timepoints. The EC50 for a recombinant VRC01-P2A-IgG standard was used to quantify VRC01 in the serum in ng/ml. **E** An IgG-specific anti-P2A ELISA was used to quantify VRC01 of this isotype in animal sera at various indicated timepoints. A standard allowed for quantification in ng/ml. **F** An HIV pseudovirus that is highly sensitive to VRC01 neutralization and that did not exhibit non-IgG based serum neutralization (001428_2_42) was used to measure the amount of functional engineered antibody in the serum using TZM-bl virus neutralization assays. Known VRC01 IC50 for this virus (0.019 µg/ml) was used to assess functional VRC01 in the serum based on SDF neutralization IC50s for each sample. All measurements for ELISA and neutralization assays were made in duplicate. Upper and lower error limits for EC50 values are indicated.

NHPs were screened for ex vivo B cell reprogramming efficiency to select three for a pilot vaccination study. Genomic sequencing confirmed intact gRNA/PAM sites in both *IgH*-alleles and minimal variation across HAs for these donors (Fig. S6). From each animal, 15-40 million (M) B cells were purified, yielding 10–22 M viable, targeted cells for infusion on day 5-6 post-activation (Fig. 3A). With editing efficiencies of 4–5%, approximately 0.5–1.2 M VRC01-expressing cells were autologously infused per animal via the saphenous vein (Fig. 3B, S7A–C). Twelve to thirteen days post-infusion, animals were primed subcutaneously in both quadriceps with MD39-ferritin [63] formulated in saponin/MPLA nanoparticle (SMNP) adjuvant [64] and boosted ten weeks later (Fig. 3A). Antigen-specific IgG titers rose to serum dilution factor (SDF) EC_50_ values of ~100 post-prime and 1-10 thousand (K) post-boost, indicating successful immunization in all animals (Fig. 3C, S8A). Large quantities of VRC01-bnAb could be detected in the serum of one animal (12093) after prime, peaking at close to 1 µg/ml, 14-days after vaccination (Fig. 3D). This titer was predominantly detected as IgG (Fig. 3E). These levels declined, however, and homologous boosting failed to re-elicit serum VRC01 in any animal (Fig. S8B). Functional VRC01 serum concentrations were calculated to be ~0.75 µg/ml on day-14, and ~0.57 µg/ml on day-21 post-prime based on 001428_2_42 pseudovirus serum neutralization, consistent with ELISA measurements (Fig. 3F). To reduce competition from endogenous memory B cells, animals received a heterologous boost at week-25 with VRC01-specific eOD-GT5-60mer (KD = 57 nM) [65] and ZM197-4mer (KD ≈ 500 nM) nanoparticles formulated in SMNP. Despite robust antigen-specific responses (Fig. S8A), VRC01-titers could not be detected after the heterologous boost in any animal (Fig. S8B).

### VRC01-cells are detected as circulating ASCs but not as GCBs in draining lymph nodes after prime

**Fig. 4:**
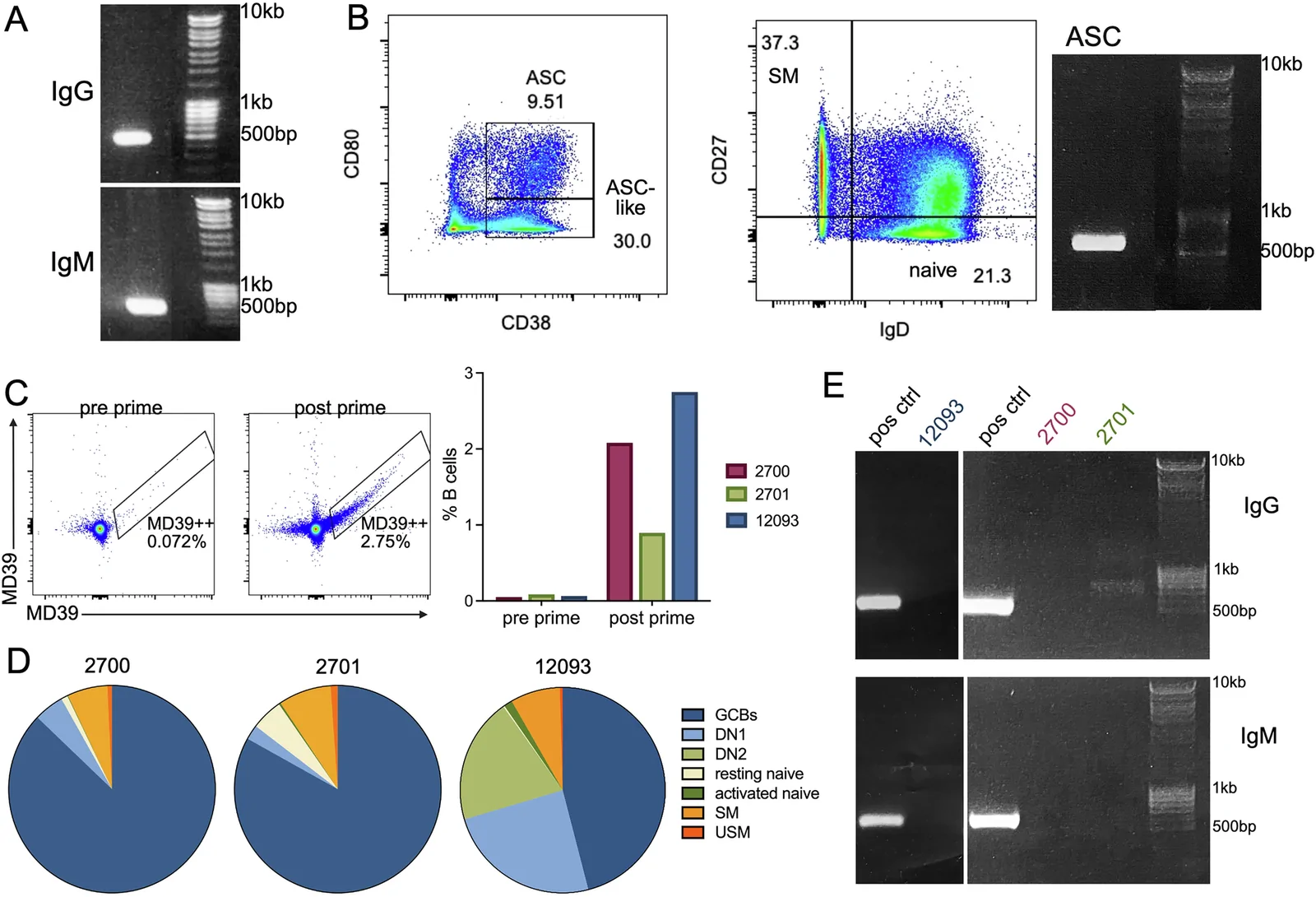
*IgH*-reprogrammed B cells are detected as ASCs in PBMCs but not as GCBs in draining lymph nodes after prime. **A** PCR amplification of VRC01 from cDNA isolated from B cells in PBMCs collected from the blood of each animal at the following timepoints; pre-engraftment, engraftment, prime, one and two weeks post prime, boost, one- and two-weeks post boost. Sequence confirmed VRC01 could be amplified exclusively in animal 12093 14-days after prime as both IgG and as IgM only (shown). **B** ASCs (CD20-,CD80 + ,CD38 + ), ASC-like (CD20-,CD80mid/-,CD38 + ), naïve B (CD20 + ,CD27-,IgD + ) and SM (CD20+ ,CD27+ ,IgD-) populations were sorted from another vial of PBMCs collected from animal 12093 14-days after prime (left) and cDNA was made from each pool of cells. VRC01 could be PCR amplified exclusively from ASC cDNA as IgG (right). **C** Example gating (animal 12093) and frequency of MD39++ B cells in biopsied draining LNs before and after MD39-ferritin prime by flow cytometry. **D** The corresponding phenotype of MD39++ B cells from (C) for each animal by flow cytometry. **E** VRC01 cannot be amplified as IgG or IgM from cDNA generated from draining LN biopsied cells as compared to cDNA from targeted primary B cells containing <1% VRC01 engineered cells (pos ctrl).

To evaluate the fate of engineered cells following vaccination, we analyzed peripheral blood mononuclear cells (PBMCs) by RT-PCR to detect transgene mRNA after immunization. Sequence confirmed VRC01 IgM and IgG transcripts were detected exclusively at day-14 post-prime in the animal with high VRC01 serum titer after prime (12093) (Fig. 4A). Day-14 PBMCs from this animal were sorted into ASCs (CD38+CD80+), ASC-like (CD38+CD80-), naïve (IgD+CD27-), and SM (IgD-CD27+) populations (Fig. S9). VRC01 as IgG was detected only in the ASC fraction (Fig. 4B). IgM-transcripts were not observed in sorted populations, possibly due to their presence in unsorted USM (IgD+CD27+) cells or low abundance, consistent with the limited circulating frequencies of engineered cells in large animals. Draining lymph nodes (LNs) from vaccinated animals were biopsied four weeks post-prime, corresponding to the expected peak of the GC response based on previous experiments [64]. Cells from these samples had high GCB frequencies relative to pre-prime controls and a substantial proportion (0.74–1.78%) were specific for MD39-trimer by flow cytometry (Fig. 4C, D). MD39-trimer reactive cells were not also eOD-GT8 reactive (VRC01-like) (Fig. S10), however, and VRC01-specific RT-PCR failed to detect transgenic antibody in these populations (Fig. 4E). Therefore, despite robust GC-induction by MD39-ferritin, no evidence of engineered B cell participation in GCs was observed. Instead, VRC01 expression was restricted to circulating ASCs, consistent with generation of a transient extrafollicular response. Interestingly, vaccination elicited a higher frequency of antigen-specific DN cells in the highest responder (12093) (Fig. 4D), suggesting a role in the induction of VRC01-ASCs in this animal.

### Activation media can be modified to alter phenotype and expand IgH-reprogrammed cells

We attempted to address several limitations of our initial pilot study, including improving the phenotype and yield of autologous *IgH*-reprogrammed B cells. We hypothesized that removing CpG from the activation media would reduce the prevalence of terminally fated DNs and ASCs while increasing the frequency of activated naïve B cells, more likely to respond productively to vaccination after adoptive transfer [66]. A cocktail containing CD40L, IL-4, IL-21, and BAFF [67] indeed maintained engineering efficiency while substantially increasing the frequency of desired B cell phenotypes by flow cytometry [67] (Fig. S11A, B). We also aimed to increase the number of engineered cells transferred, since the ratio of VRC01-B cells engrafted to animal body weight was 20-40-fold lower for the NHP pilot compared to our successful murine model [44]. We optimized an ex vivo expansion strategy using hCD40L-expressing feeder cells that enabled affordable, low-density B cell culture with IL-4, IL-21, and BAFF. Cells were cultured for 6-days post-engineering, achieving 5-13-fold expansion, while preserving a predominantly activated naïve B cell phenotype (Fig. S11C). To avoid co-transferring feeder cells into animals, VRC01-cells were enriched using magnetic-activated cell sorting (MACS) with biotinylated eOD-GT8 and releasable streptavidin beads for one passage with soluble CD40L prior to infusion (Fig. S11D).

### Engraftment of expanded IgH-reprogrammed cells into vaccinated animals

**Fig. 5:**
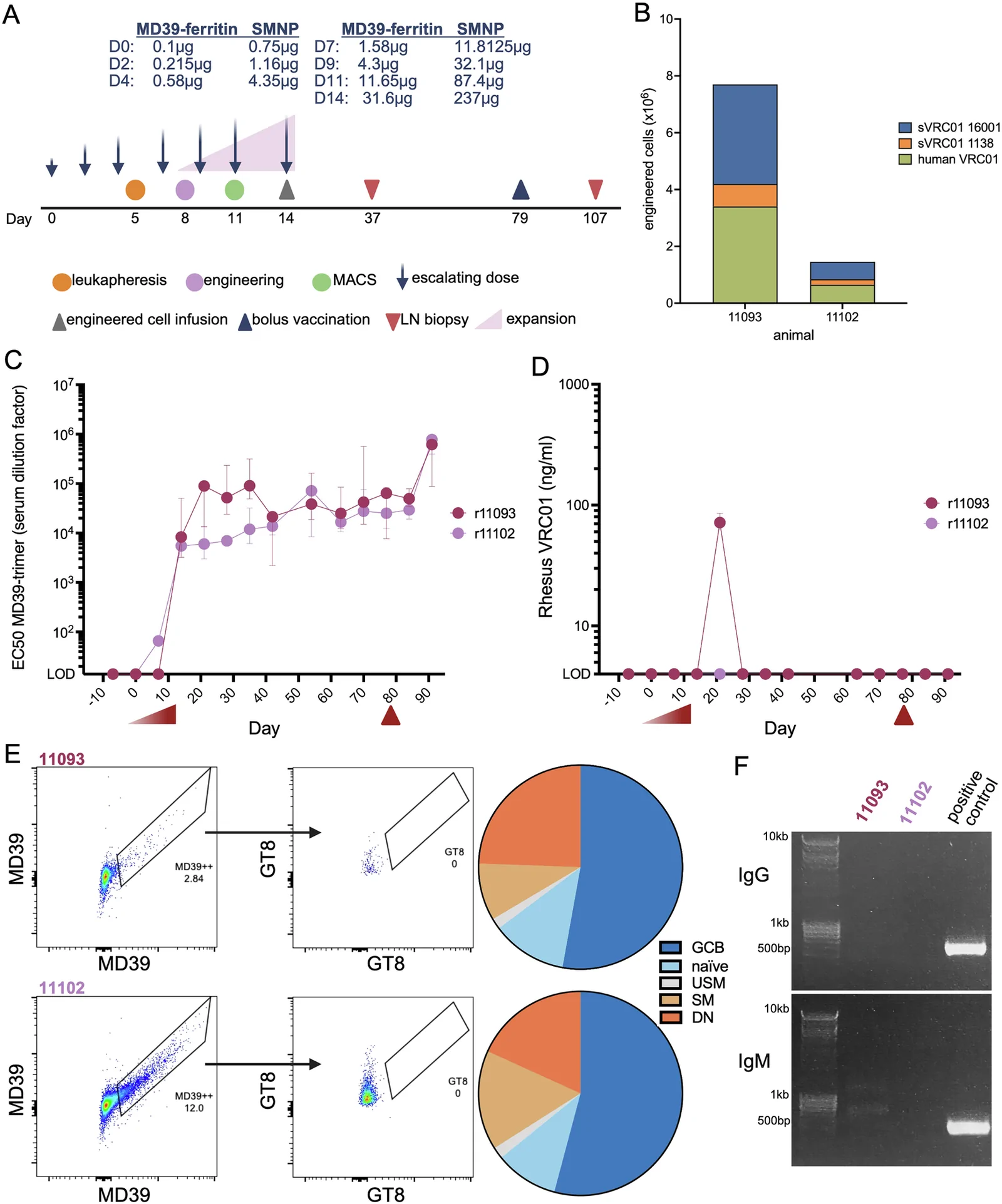
Experimental setup and results of a second pilot transfer and vaccination experiment. **A** Timeline for reprogrammed B cell engineering and transfer, vaccination, and sampling in pilot-2. Blood draws were performed weekly for serum, PBMC, and plasma collection. Escalating dose prime was performed as indicated above the experimental timeline (values given are for one dose, and two doses were administered to four sites). **B** Number of VRC01 and sVRC01 engineered cells engrafted. **C** Total MD39-trimer-specific IgG titers generated by MD39-ferritin vaccination were assessed by ELISA and are given as SDF EC50s. Red triangles indicate escalating dose prime and bolus boost. Titers are 100-1000x higher after prime compared to pilot-1. **D** An anti-P2A ELISA was used to quantify total VRC01antibody in animal sera at indicated timepoints. Red triangles indicate escalating dose prime and bolus boost with MD39-ferritin. Roughly 100 ng/ml of VRC01 could be elicited in 11093 after prime, peaking 1-week after adoptive cell transfer before rapidly declining. **E** Frequency of MD39 + + and MD39 + +/eOD-GT8 + + (VRC01-like binding) B cells in draining LNs collected 4-weeks after the first dose of the MD39-ferritin prime (left) and phenotype of MD39 + + cells at the same timepoint (right). No (s)VRC01 cells could be detected in VRC01-like flow cytometry gate. **F** (s)VRC01 cannot be amplified as IgG or IgM from cDNA generated from draining LN biopsied cells from either donor as compared to cDNA from targeted primary B cells containing 0.5% VRC01 engineered cells.

With improved cell culture protocols, we conducted a second pilot study in two animals. We included HRTs encoding two “simianized” versions of VRC01 (sVRC01) to reduce the likelihood of host immune clearance of engineered cells (Fig. S12). To engage expanded, engineered cells in an activated state, we adoptively transferred them at the end of an escalating-dose MD39-ferritin vaccination designed to enhance GC-participation (Fig. 5A). This regimen, administered subcutaneously over 2-weeks, has been shown to increase antigen-specific GCB responses >10-fold by improving native immunogen deposition on follicular dendritic cells (FDCs) and T follicular helper cell (Tfh) generation [68, 69]. Animals were leukapheresed and purified B cells were engineered with (s)VRC01 AAV donors and cultured in expansion media. Between 2-8 M (s)VRC01 cells were autologously infused back into donor animals on the final day of vaccination (Fig. 5B). A homologous bolus boost was given 10 weeks after cell transfer. MD39-trimer-specific serum IgG titers rose to SDF EC_50_ values between 10 and 100 K after prime, roughly 100-1K-fold higher than responses to bolus immunization (Fig. 3C), and to ~1 M after boosting (Fig. 5C). Unfortunately, VRC01 titers in serum resembled that of pilot-1, with a burst in titer in one animal after prime, and no measurable titer in either animal after boosting (Fig. 5D). Since cell engraftment and vaccination were simultaneous in pilot-2, it was unclear if low VRC01 serum titers detected shortly after adoptive transfer were due to activation culture or engagement by vaccine immunogens in vivo. A robust endogenous response to MD39-ferritin vaccination could be observed four weeks after prime in the draining LNs from pilot-2 animals. However, MD39-specific B cells that were also reactive with GT8 could not be detected (Fig. 5E) and (s)VRC01 cDNA could not be amplified from these populations (Fig. 5F), indicating a lack of detectable *IgH*-reprogrammed cell participation in GC reactions at this timepoint.

#### Characterization of *IgH*-reprogrammed cells after in vitro differentiation to ASCs

**Fig. 6:**
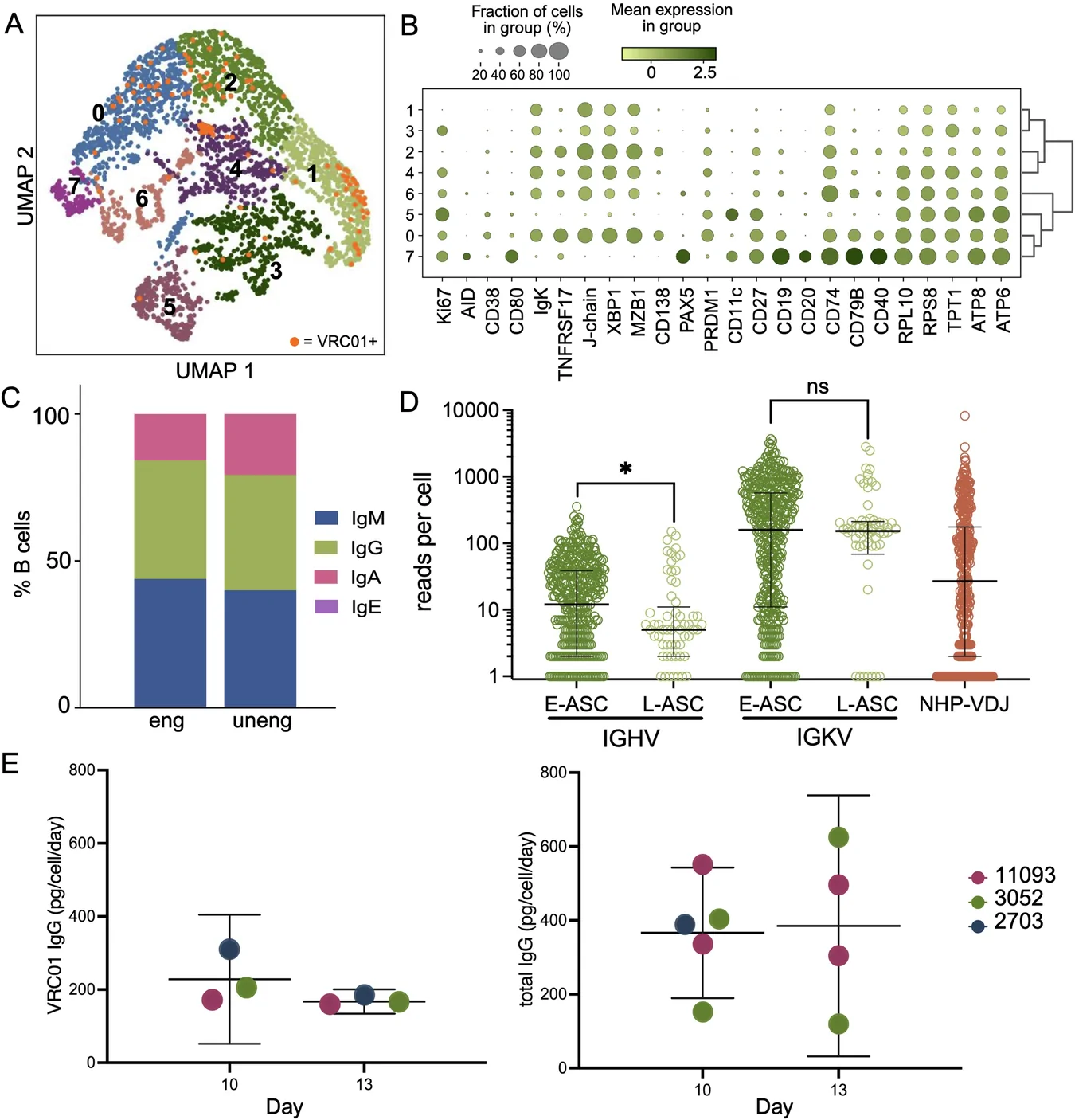
Characterization of in vitro differentiated *IgH*-reprogrammed ASCs. **A** To characterize engineered cells as ASCs generated in vitro, cells were cultured, targeted, and expanded for 13 days in ImmunoCult B cell expansion media. Engineered cells in this media were too fragile to enrich by FACS and therefore maintained a level of ~2% of cells in culture. UMAP Leiden clusters of single cell RNA transcriptomes are shown, with engineered cells identified as orange dots. **B** Relative transcriptional levels for genes of interest from cells divided by cluster. **C** Comparison of isotype in engineered vs unengineered cells, as determined by analysis of *IgH* constant gene transcripts in scRNAseq data. **D** Number of sequencing reads per engineered ASC that aligned to either VRC01 IGHV or IGKV genes after 6 days in pilot-1 media (“early” E-ASCs) or after 13-days in ImmunoCult (“late” L-ASCs). Unengineered cell IGHV gene transcript read numbers after 13-days in ImmunoCult are shown for comparison (orange). Medians with interquartile ranges for each data set are indicated. Significant differences between the indicated groups were assessed by Mann-Whitney t-tests. **E** VRC01 IgG (left) and total IgG (right) secreted from a pool of engineered cells (~2% VRC01 + ) in culture on day -10 and day-13. VRC01 specific and total IgG were determined by P2A-specific ELISA using cell culture supernatants from each timepoint. IgG values were then divided by cell number and time in culture to calculate a final concentration in pg/cell/day.

To further characterize our *IgH*-reprogrammed cells as in vitro differentiated ASCs, engineered B cells were cultured in ImmunoCult media (Fig. S13A, B). By day 13, 50% of total cells and 65–80% of VRC01+ cells expressed ASC markers (CD20-CD38+CD80+) by flow cytometry (Fig. S13C). scRNA-seq showed VRC01+ cells clustering mostly as ASCs (clusters 0-4 and 6) based on differential gene transcription. Of these, only clusters 1 and 2 appear to have exited the cell cycle, based on Ki67 levels. Other populations include transitional memory PCs (clusters 5), and a small subset of activated B cells (cluster 7) (Fig. 6A, B). Both engineered and unengineered ASCs underwent class switching to IgG and IgA (Fig. 6C, Fig. S13D), and a second donor showed similar results (Fig. S14). Reprogrammed B cells grown in ImmunoCult were too fragile to enrich by FACS and were thus at low frequencies in scRNA-seq data. VRC01-transcripts in these “late” ASCs (L-ASCs) trended slightly lower relative to “early” ASCs (E-ASCs) from 6-days in pilot-1 media, however, little difference in the median or geometric means were observed. This could suggest a survival disadvantage for PCs that produce excessive levels of transgene. Unengineered L-ASCs had IGHV-transcript read numbers that were broadly and evenly distributed over several logs (Fig. 6D). VRC01-IgG secretion was quantified by ELISA and ELISPOT in L-ASC cultures, and it was estimated that engineered cells secreted 164–308 pg/cell/day, comparable to total IgG secretion (152–624 pg/cell/day) (Fig. 6E, Fig. S13E, F). These findings confirm that *IgH*-reprogrammed NHP B cells can differentiate into functional ASCs with robust transgene upregulation and antibody production in vitro, however, engineered cells with modest VRC01-transcription levels may have a survival advantage compared to high transcribers.

### NHP secondary lymphoid organoids can be used to study IgH-reprogrammed B cell responses in vitro

**Fig. 7:**
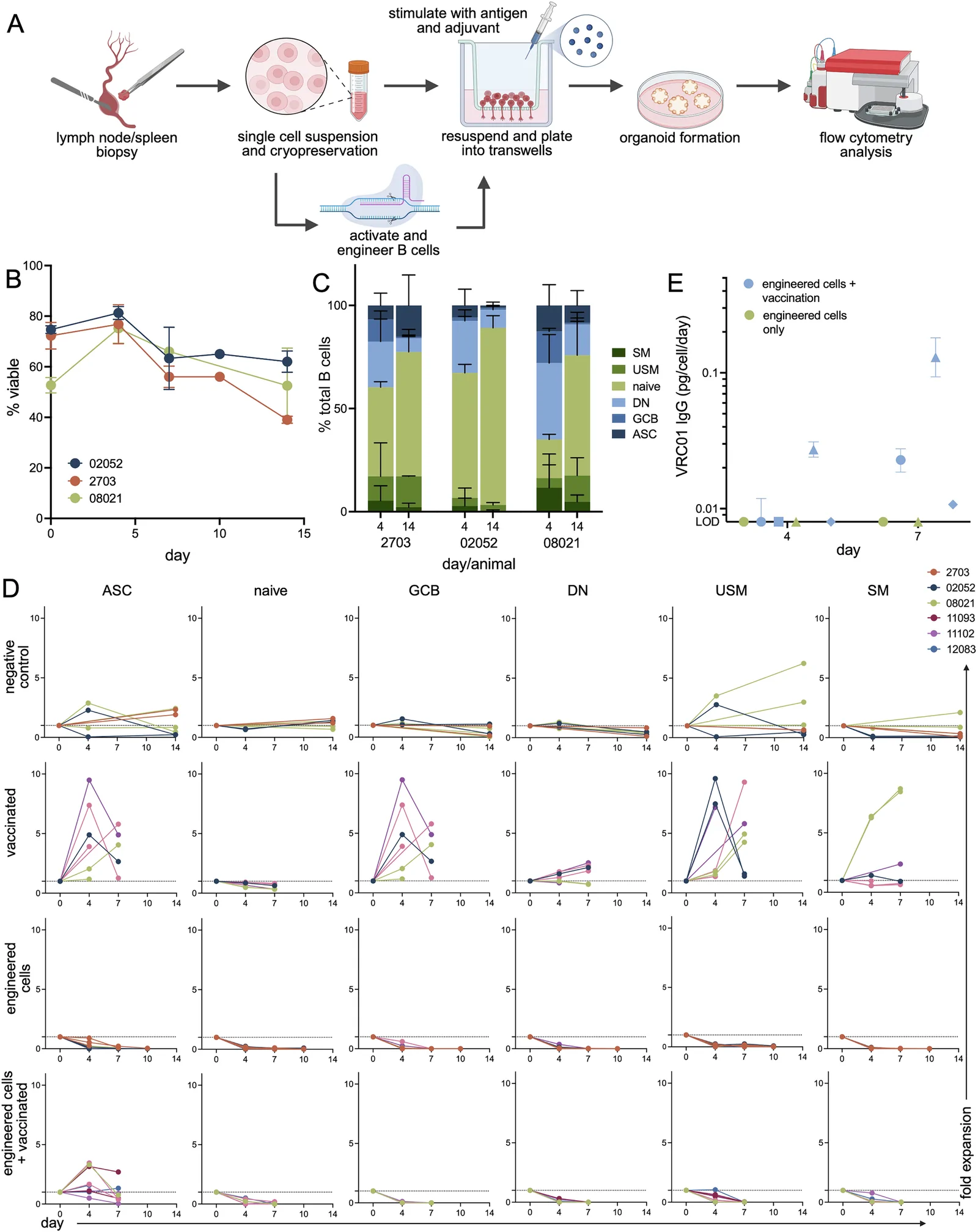
Organoids derived from secondary lymphoid tissue can be used to evaluate engineered cell responses to HIV Env based immunogens. **A** Schematic of organoid culture steps, where cryopreserved cells from biopsied LNs or spleens can be resuspended in media containing BAFF andinsulin/selenium/transferrin supplement and cultured in transwells. Engineered cells can be added to organoids at the time of culture to evaluate survival and phenotype over time. In addition, organoids can be vaccinated on the first day of culture (day-0) to evaluate endogenous and engineered responses. **B** Viability of all single cells in organoid cultures at the indicated time points over a 14-day period (*n* = 2–4 where error bars are present). Error bars represent standard deviation from the mean. **C** Phenotype of organoid B cells on day 0 and 14 of culture for three representative animals (*n* = 2–3 replicates per animal; error bars represent standard deviation). **D** (From top to bottom) Fold expansion of B cell phenotypes over time in negative control organoid (top row), organoids vaccinated with 1 µg/ml MD39-ferritin and SMNP (second row), engineered cells cultured in organoids (third row), and engineered cells cultured in organoids and vaccinated with 1 µg/ml MD39-ferritin and SMNP (bottom row). **E** Quantification of VRC01 IgG secreted by engineered cells with or without MD39-ferritin vaccination, as determined by anti-P2A ELISA. Each symbol represents a different donor, *n* = 2 for each donor, and error bars represent standard deviation from the mean. LOD is the lower limit of detection.

We wanted to characterize the survival, phenotype, and in vivo behavior of *IgH*-reprogrammed B cells after adoptive transfer and vaccination in NHPs; however, they are relatively inaccessible in these large animals. We hypothesized that placing engineered cells into autologous SLO cultures might allow us to model their in vivo fate after autologous transfer and vaccination. It was previously shown that human tonsil, spleen, and LN tissue can be cultured to develop a functional organotypic system that recapitulates key GC features in vitro [70–72]. We were able to successfully reproduce these cultures using NHP LN and spleen biopsies (Fig. 7A) following previously established methods for human cells [70, 71]. Cultured NHP organoids could survive for at least 14-days without B cell activation supplements (Fig. 7B), and frequencies of B cell subtypes over this period were similar to those published for human organoids [70] (Fig. 7C, D).

We engineered cells in pilot-1 activation media and autologously combined them with biopsied LN/spleen cells to create organoids. Engineered cells were difficult to detect beyond 7-10 days in culture (Fig. 7D, Fig. S16A). Consequently, the identity of the engineered B cell population that responded to immunization in vivo remains unclear. These findings suggest that further optimization of organoid cultures may be required to more accurately model the fate of engineered cells post-transfer, especially since pilot-1 data show that some engineered cells persist and respond to vaccination.

Vaccination of organoids at culture initiation with MD39-ferritin formulated in SMNP adjuvant, however, induced significant expansion of engineered cells as ASCs within 4–7 days (Fig. 7D). This was accompanied by detection of VRC01 antibody secreted into culture organoid supernatants (Fig. 7E). Engineered cells could not also be detected as GC-like B cells despite robust elicitation of GCB-like endogenous responses in the vaccinated organoids. This in vitro response mirrored the transient, ASC-dominated fate of VRC01-reprogrammed cells observed in vivo. Notably, the magnitude of engineered responses varied by donor, suggesting donor intrinsic differences in engineered B cell potential. These results suggest that NHP-derived SLOs may offer a valuable platform to optimize engineering or vaccination protocols intended to elicit GC-derived, durable transgenic bnAb memory responses in NHPs in vivo.

## Discussion

This study represents a significant advance in the translation of engineered B cell vaccines to clinically relevant NHP models. We demonstrated that modest numbers of *IgH*-reprogrammed cells expressing the VRC01-bnAb can survive autologous infusion, traffic to secondary lymphoid tissues, and respond to subcutaneous vaccination with HIV Env immunogens. Following prime immunization with MD39-ferritin, engineered cells could differentiate into circulating ASCs capable of producing high, isotype-switched serum VRC01-titers. However, these titers were donor specific, transient, and unboostable, with no evidence of GC-maturation or the generation of durable memory or LLPCs, consistent with extrafollicular differentiation.

This outcome contrasts sharply with results obtained using the same transgenic BCR and immunogen combination in mice, where *IgH*-reprogrammed cells predominantly and consistently undergo GC maturation, generating boostable memory and PCs that sustain durable serum VRC01 titers [44]. Importantly, key differences in how these models are implemented point to specific, testable explanations for the divergent outcomes and provide a framework for optimizing next-generation NHP studies.

First, differences in the ex vivo activation conditions required for B cell engineering across models (TLR4-based activation in mice versus CD40-based activation in NHPs) may result in distinct in vivo behaviors following adoptive transfer. Although direct characterization of rare, engineered cells after transfer is technically challenging in large-animal models, defining their trafficking, survival, and differentiation in vivo as has recently been demonstrated for in vitro differentiated PCs [73] could reveal critical features of *IgH*-reprogrammed B cells in the NHPs that limit their participation in GCs. Such insights would directly inform the optimization of activation strategies better suited for primate studies.

Second, despite development of an NHP B cell expansion step, the number of engineered cells transferred into recipient animals remained low compared to what can be achieved in mice. Increasing *IgH*-targeting efficiencies may therefore contribute to improved robustness and reproducibility of engineered responses to vaccination in NHPs. Notably, targeting efficiencies approaching 40% have recently been reported using small HRTs that insert promoter-free bnAb cassettes into the *IgH*-locus of NHP B cells as exons, relying on endogenous IGHV-gene promoters for transcription [48].

Third, intrinsic differences between engineered mouse and primate B cells may contribute to the divergent in vivo behaviors observed across species. Deeper characterization of engineered mouse B cells using scRNA-seq and the employment of calcium flux BCR-activation assays in engineered mouse and NHP B cells may reveal important differences in BCR expression, cell activation potential, or responsiveness to T cell help. This may inform the selection of alternative immunogens and adjuvants that more faithfully drive GC-entry, memory formation, and LLPC differentiation in NHPs.

Finally, colocalization of intravenously infused engineered B cells with vaccine-induced immune responses is inherently less efficient in large animals compared with mice. This limitation may be addressed by directly injecting engineered cells into immunized lymph nodes, developing vaccines that elicit robust splenic responses, or transiently expressing lymphoid homing receptors, such as CXCR5 and CCR7 during the engineering process. More broadly, strategies that co-deliver in vivo *IgH*-reprogramming reagents alongside vaccine primes may maximize colocalization of engineered B cells with antigen and cognate Tfh, while bypassing limitations imposed by ex vivo activation on persistence, homing, or function.

To facilitate optimization of the NHP model, we developed an NHP SLO culture system and showed that vaccination of autologous VRC01-expressing B cells with MD39-ferritin in this setting recapitulates the transient, donor-variable ASC responses observed in vivo. This finding highlights the potential utility of NHP SLOs as an in vitro platform for screening immunogen and adjuvant combinations, activation conditions, and in vivo engineering strategies prior to undertaking lengthy and costly in vivo studies. Moreover, interrogation of engineered B cell interactions with Tfh cells and other accessory populations within these organoids may provide mechanistic insight into the extrafollicular bias observed in early NHP experiments.

Together, these findings demonstrate that ex vivo IgH-reprogrammed B cells can survive and respond to vaccination in NHPs, and that the extrafollicular responses observed in vivo in this study could be recapitulated in a novel NHP SLO culture system. This work therefore establishes a foundation for the design of “engineered B cell vaccines” that elicit durable bnAb memory responses in NHPs, advancing such a cell therapy approach toward clinical translation as a potential functional cure for HIV.

## Methods and Protocols

### Study design

This study aimed to evaluate the capacity of genome-edited B cells expressing HIV bnAbs to engraft, persist, and respond to Env vaccination in NHPs, and to model these responses in vitro using SLO cultures. Two small, exploratory adoptive cell transfer and vaccination experiments were conducted across a total of five animals. These pilot studies were not designed to be statistically powered but were intended to generate preliminary data to guide the refinement of protocols and design of larger, hypothesis-driven experiments. Investigators were not blinded during sample analysis.

### Ethics statement

Indian rhesus macaques (*Macaca mulatta*) were housed at the Wisconsin National Primate Research Center (WNPRC). Animals were monitored twice daily by veterinarians for any signs of disease, injury, pain, distress, or psychological abnormalities and treated as recommended by the veterinarian. Involved animals were not euthanized and were returned to the colony at the end of the study. All methods were performed in accordance with relevant guidelines and regulations and were approved by the University of Wisconsin-Madison College of Letters and Sciences and the Office of the Vice Chancellor for Research Institutional Animal Care and Use Committee (IACUC protocol number G006242). The animal facilities of the WNPRC are licensed by the US Department of Agriculture and accredited by AAALAC.

### Leukapheresis

Large numbers of PBMCs were collected from each animal by leukapheresis. The apheresis was performed using a Spectra Optia apheresis unit (Terumo BCT) primed with autologous blood. Animals were sedated during the apheresis procedure, and catheters were placed in the femoral vein for collection and the saphenous vein(s) to return blood to the animal. The animal’s vital signs were monitored throughout the procedure (e.g., heart and respiratory rate, ECG, oxygen saturation, blood pressure, temperature, etc.). Calcium and other electrolytes were also monitored, and calcium levels adjusted with calcium gluconate as needed to maintain baseline calcium levels. Blood was processed by Ficoll-Paque Plus (Cytiva 17144002) density gradient separation for PBMCs. PBMCs were counted and aliquots frozen in CryoStor CS10 (Stem Cell 07930).

### Blood, tissue, and cell collection

Ten milli liters of blood was collected into EDTA-coated vacutainer tubes (BD Biosciences 367863) for separation into plasma and PBMCs using Ficoll-Paque Plus (Cytiva) density gradient separation. Aliquots of plasma were stored at −80 °C, and PBMCs were resuspended in CryoStor for storage of aliquots at <-150 °C. 5 ml of blood was collected into Serum Separation Tubes (SSTs) to prepare serum by centrifugation. Aliquots were stored at −80 °C for use in ELISA and pseudovirus neutralization assays. Serum was heat inactivated at 42 °C for 30 min before use in assays. Lymph node biopsies were collected from an inguinal or axillary sites four weeks after prime, and one week after each boost depending on the site of vaccination. Lymphocytes were isolated by dicing the tissue and pressing it through a 70 µM cell strainer. Cells were washed in R10 media (Gibco), counted, aliquoted and frozen in CryoStor media for storage at < -150 °C.

### B cell infusions and vaccinations

(s)VRC01 targeted B cells were analyzed by flow cytometry to assess engineering efficiency, resuspended 5 ml of L-15 Leibovitz sterile tissue culture media, and autologously infused into each animal. Sedated animals were fitted with a catheter into the saphenous vein and cells were slowly infused intravenously over the course of approximately one hour while being monitored by a veterinarian. In pilot-1, 12-13 days after infusion, animals were primed and then boosted subcutaneously 10 and 25 weeks after priming with endotoxin-free recombinant protein immunogens formulated with SMNP adjuvant. MD39-ferritin [63] or eOD-GT5-60mer [74] and ZM197-4mer recombinant proteins were provided by the laboratory of Dr. William Schief and SMNP was prepared as previously described by the laboratory of Dr. Darrell Irvine [64]. In pilot-1, each dose was 100 µg of protein prepared with 750 µg of SMNP as previously described [64]. Bolus subcutaneous vaccination dosages were split in half and administered bilaterally into the quadriceps. For heterologous boost, 50 µg each of eOD-GT5-60mer and ZM197-4mer was mixed with SMNP and divided between the two vaccination sites. In pilot-2, animals were primed with an escalating dose regimen of MD39-ferritin with SMNP, as previously described [69]. Briefly, two doses of prime prepared as above were administered subcutaneously at four sites (bilaterally into quadriceps and biceps) according to the schedule given in Fig. 5. Two doses of homologous boost were administered as a bolus to these same sites 10w after prime.

### Cell lines

293-T and TZM-bl cells were obtained from the American Type Culture Collection (ATCC). They were cultured in DMEM (Corning) with 10% FBS (Cell Culture Collective), 1% L-glutamine, and 1% penicillin/streptomycin (P/S) (Gibco). Expi293 cells (Gibco) were used for monoclonal antibody standard production. They were maintained in Expi293 media (Gibco) according to the manufacturer instructions. A VRC01-engineered mouse pro B cell line was used as a positive control for GT8^++^/MD39^++^ flow cytometry gating of VRC01-reprogrammed NHP B cells. This line was maintained in RPMI (Gibco) with 10% FBS, 1% L-glutamine, 1% NEAA (Gibco), 1% Na Pyruvate (Gibco), and 1% P/S. 3T3-fibroblasts expressing CD40L were provided by the laboratory of Dr. Gina Doody [75]. These cells were cultured in IMDM (Gibco) supplemented with 10% FBS (Cell Culture Collective), 1% L-glutamine, 1% non-essential amino acids (NEAA), 1% Sodium Pyruvate, 1% P/S. To irradiate, cells were detached from tissue culture flasks using 10 ml Trypsin/EDTA, then 15 ml of fresh media was added (2.5-3.5 ×107 cells per T150 flask), and cells were irradiated on ice with 50 Gy for 45 min. After irradiation, cells were washed in culture media and aliquots were frozen down in IMDM with 10% FBS and 10% DMSO for storage at -80°C.

### Primary B cell isolation and culture

Frozen PBMCs were thawed and treated with DNase-I (StemCell, 07900) for 15 min before proceeding onto B cell isolation through negative selection with magnetic beads (StemCell, 100-0345) according to the manufacturer’s instructions. Pilot-1 and -2 culture media used the following base media: Iscove’s Modified Dulbecco’s Medium (IMDM) (Gibco), supplemented with 10% FBS (Cell Culture Collective), 1% L-glutamine, 1% MEM Non-Essential Amino Acids (NEAAs), 1% Sodium Pyruvate, 1% P/S and 0.1% ß-mercaptoethanol (BME) (Gibco). Media could be stored at 4°C in the dark for two months. Activation supplements were added directly before use. Pilot-1 activation supplements included 100 ng/mL recombinant human MEGACD40L (Enzo, ALX-522-110-C010), 2.5 µg/mL CpG ODN2006 (Adipogen, ODN2006-1), 50 ng/mL IL-2 (Peprotech, 200-02), 10 ng/mL IL-4 (Miltenyi, 130-093-922). Cells were plated at a density of 1–1.5 × 10^6^ cells/ml and cultured for three days before changing media. To change media, cells were centrifuged at 400 g for 5 min and the pellet resuspended in fresh activation media, at a density of 1–1.5 × 10^6^ cells/ml. Cells could be cultured this way for 9 days. Pilot-2 media included 50 ng/mL IL-4 (Miltenyi), 8 ng/ml IL-21 (Biolegend,130-093-922), and human BAFF (Biolegend, 559602). Cells were plated at a density of 0.5 × 10^6^ cells/ml in tissue culture plates previously seeded with irradiated 3T3-human CD40L (hCD40L) feeder cells [75] at 0.5 × 10^6^ cells/ml. After three days, cells were engineered and resuspended in fresh media and on new 3T3-hCD40-feeder cells. After 3 more days of expansion culture, cells were removed from feeder cells and cultured for a final passage at 0.5 × 10^6^ cells/ml in media containing 100 ng/mL recombinant human MEGACD40L (Enzo) in place of feeder cells. For ImmunoCult cultures, isolated B cells were cultured using a human B cell expansion kit (StemCell, 100-0645). Cells were cultured at a density of 3–5 × 10^5^ initially, and subsequently at 1.5 × 10^5^ after engineering for optimal expansion. Cells were placed in fresh media every 3 days and could be cultured for up to 14 days. In all cases, cells were grown at 37 °C, with 5% CO2 and 95% humidity. To assess the mitogenic properties of activation media, 1 million B cells were stained in 5 µM of Carboxyfluorescein Succinimidyl Ester (CFSE) (Thermo, C1157) for 5 min- before placing into culture. CFSE mean florescence intensity (MFI) was assessed by flow cytometry every 24 h to monitor cell division.

### Optimization of B cell nucleofection

Primary B cells were activated in culture for 24–48 h before being subjected to the T and R buffer nucleofection optimization experiments for the Neon (Invitrogen, MPK1096), according to the manufacturer’s instructions. Briefly, 0.5 M cells were washed and resuspended in 10 µl of R or T buffer containing 0.5 µg of GFP reporter mRNA (TriLink, L-8101-100). The nucleofection buffer (R/T) was never less than 80% of the total volume. Immediately after electroporation in a 10 µl Neon tip, the cells were recovered in Pilot-1 culture without antibiotics. After 2 hr, antibiotics were added back to the media. Cell viability and GFP expression was assessed by flow cytometry 24–48 h later. Electroporation conditions that balanced cell viability with high GFP expression were selected for B cell engineering.

### Design and testing of IgH-targeting guide RNA

spCas9 gRNAs targeting the *IgH*-locus were designed using Horizon Discovery CRISPR Design Tool. Candidates with 20 nucleotide guide sequences were ordered from IDT as crRNA and complexed with trRNA (IDT) at a 1:1 mass ratio according to manufacturer instructions. Ribonucleoprotein (RNP) was prepared by complexing the crRNA/trRNA with spCas9 protein (IDT, 10007803) at a mass ratio of 1:1.2 and incubating the mixture for 20-30 min before electroporation. Electroporation Enhancer (IDT, 10007805) was added after the 20-30 min incubation, according to manufacturer instructions. After three days of culture in pilot-1 media, 3-5 × 10^5^ cells were washed and resuspended in 10 µl R buffer containing 1 µl of RNP and electroporated with one 20 ms pulse at 1650 V in a 10 µl Neon tip. Cells were immediately recovered in activation media without antibiotics at a density of 1 × 10^6^ cells/ml. As a control, cells were electroporated without RNP using the same conditions. Two days later, genomic DNA (gDNA) was isolated using the DNeasy kit (Qiagen). The gDNA region flanking the gRNA cut site was amplified by PCR using the following primers: 5’-AGGCCGACAGTGGTCTGGC-3’ (F) and 5’-GACACATTCC TCAGCCATCA-3’ (RC). The resulting PCR product was purified using a MinElute reaction cleanup kit (Qiagen) and Sanger-sequenced (Eton). The frequency of indels in RNP electroporated cells relative to control electroporated cells was determined using TIDE [76].

### bnAb donor DNA design and production as AAV

VRC01 donor DNA was designed as follows from 5′ to 3′: (1) 554-bp HA matching sequence upstream of the Macaca mulatta *IgH-J6* RNP cut-site generated by the 20 bp gRNA sequence (5’-GTCGTCGTCACCGTCTCCTC-3’), (2) rhesus IGHV4-AFI*01 gene promoter region (1500 bp 5′-UTR), (3) rhesus IGKV1-AAY*01 leader (intron removed) and mature human VRC01 kappa light-chain variable region, followed by the Macaca mulatta IGKC without stop codon, (4) GSG linker followed by a P2A [77] self-cleaving peptide sequence, (5) rhesus IGHV3-AFR*01 leader (intron removed) and mature human VRC01 heavy-chain variable region, (6) 540-bp HA downstream of the *IgH-J6* RNP cut site. A splice donor consensus sequence was preserved at the VDJ-homology arm junction. The HAs and V-gene promoter were amplified from Indian rhesus macaque genomic DNA (gDNA) using the following primer sets : 1) 5’HA 5’–TGGAGGCACTTTGGAGGTCAGGAAA-3’ (F) and 5’-GAGACGGTGACGACGACCCCTTG GCCCCAGGAATCCA-3’ (RC), 2) IGHV4-AFI 5’-CCACCTGCCATATTTGCGGCAAACAATAC-3’ (F) and 5’-GTTCTTGCACAGGATGTCCGTGAC-3’ RC, 3’HA – 5’-GTAAGAATGGCC ACTCTAGAGCC-3’ F and 5’-CTCTTGGAAACCAACTTCAGGGCAC-3’ RC. Macaca mulatta HRT elements (gRNA, HAs, promoter, and leaders) were based on GenBank IGH reference MF989451.1. The VRC01 open reading frame was synthesized as a single gene (GeneArt) and all DNA fragments were assembled using Gibson assembly (NEB) into a pITR AAV backbone (provided by the laboratory of Dr. Paula Cannon) according to the manufacturer instructions and transformed into chemically competent K12 *E. coli* (NEB). Single colonies from bacteria plated on agar containing ampicillin were cultured. Plasmids were isolated (Qiagen) and the entire plasmid sequenced (Eton Biosciences or Plasmidsaurus). The VRC01 pITR vector was produced as the AAV6 serotype (Vector Biolabs) and stored as aliquots at −80 °C. To create HRT-AAVs with new bnAb specificities, the bnAb LC (with Macaca mulatta IGKC or IGLC*03 constant region), GSG linker, P2A peptide, bnAb HC variable region, and the first 60 bp of intronic region corresponding to the bnAb *IGHJ* gene (to preserve the splice donor site) was synthesized as a single gBlock (IDT) and used for cloning between the promoter and 3’ HA of the VRC01 pITR plasmid, through Gibson Assembly. Leaders corresponding to the bnAb HC and LC germline V-genes were used for each HRT design. For RHA1.V2.01 HC only design, a gBlock encoding only the bnAb HC with leader and intron region was used, omitting the bnAb LC, LC constant gene, GSG linker and P2A peptide from the design. The natural VRC01 (Genbank: GU980703.1 and GU980702.1), RHA1.V2.01 (Genbank: MT610892.1 and MT610888.1) and Ab1482 [52] variable region codons were used.

### Design of simianized VRC01 bnAb donor templates

The mature VRC01 IGHV amino acid sequence was aligned to Macaca mulatta IGHV-genes [78]. 10 versions of VRC01 HC were made by manually replacing the human IGHV gene with NHP ones while conserving residues known to be important for antigen binding. A previously designed simianized VRC01 KLC [79] was paired with HC designs for expression as monoclonal NHP IgG recombinant proteins. Proteins were purified and tested in binding and neutralization assays before down selecting two variants for adaptation to HRTs based on expression levels and antigen binding properties.

### B cell engineering

B cells were purified and cultured for three days in activation media as previously described. 67 h after placing cells into culture, they were washed and resuspended in serum-free IMDM at a concentration of 1×10^5^ cells/µl and AAV6 bnAb-HRT (1-2×10^12-13^ genome copies (gc)/ml) (Vector Biolabs) was added at an MOI of 20,000 gc/cell. Cells were transduced with AAV for 1.5 h at 37 °C in 1.5 ml Eppendorf tubes for small volume reactions (< 3 million cells), or 50 ml conical for large volume reactions (> 3 million cells) before electroporation using the Neon nucleofection system (Invitrogen, MPK10096) to introduce CRISPR-Cas9 RNP. A 50 ml conical is used for large scale reactions to accommodate adding media for spinning down, adding enough R buffer volume and using the Neon pipette (15 ml conical is too narrow to fit Neon pipette). After transduction, 1 ml IMDM was added, and cells were spun down at 400 g for 5 min and AAV-containing supernatants were added to recovery media (activation media without antibiotics). If <1 M cells were used, spinning was omitted, and we proceeded directly with the Neon reaction. Up to 5×10^6^ cells were then resuspended in R buffer with 10 µl RNP for nucleofection in a 100 µl neon tip using one 20 ms pulse at 1650 V. RNPs were prepared with Electroporation Enhancer as described above. R buffer was never less than 80% of the total volume in the tip. For small-scale transduction reactions of ~0.5×10^6^ B cells, R buffer and RNP were directly added to cell pellets for electroporation in 10 µl neon tips. Cells were recovered immediately after nucleofection. Recovery media volume was always >10-fold more than the neon tip volume to sufficiently dilute the R buffer. Cells cultured in pilot-1 media were recovered at 1 ×10^6^ cells/ml, and cells cultured in pilot-2 media were recovered at 0.5×10^6^ until media change and expansion on day 6. Cells cultured in ImmunoCult media were recovered at a density of 3–5 × 10^5^ for three days and subsequently cultured at 1.5×10^5^ after engineering for optimal expansion from day 6 onward.

### Engineered cell isolation

For pilot-2, engineered cells were isolated on day 6 of culture (3 days after engineering) for subsequent expansion. Feeder cells along with B cells were released from cell culture plates, with 2 µM EDTA/trypsin. Cells were then spun down at 400 g for 5 mins before proceeding with EasySep™ Release Human Biotin Positive Selection Kit (StemCell). The kit was used according to manufacturer instructions with 0.25 µg/ml of biotinylated eOD-GT8 monomer and 0.25 µl/ml of selection cocktail.

### Neutralization assay

Pseudovirus production and neutralization assays were performed under sterile BSL2/3 conditions as previously described [80]. To produce pseudovirus, 293 T cells were plated with DMEM +  10% FBS  +  1% P/S  +  1% L-glutamine (supplemented DMEM) to achieve 50–80% confluency in 15 cm tissue culture plates (Corning, 430599) at the time of transfection. The following day, 293 T cells were co-transfected with PSG3 plasmid and pseudovirus envelope (full length) plasmid using FuGENE (Promega) and Opti-MEM transfection medium (Gibco) to produce single-round of infection-competent pseudoviruses as previously described [81]. To assess serum neutralization, 10 μl of freshly thawed, virus was diluted appropriately in supplemented DMEM and immediately mixed with 10 μl of serially diluted (2×) serum (5x starting dilution) in 384-well white plates (Corning, 3570) and incubated for 45 min at 37 °C to allow for antibody neutralization of the pseudovirus. Serum dilutions were made with supplemented DMEM and a no serum infectivity control was included. After 45 min, 3000 TZM-bl cells/well (in 20 μl of media containing 40 μg/ml Dextran) were directly added to each well containing the antibody/virus mixture. Plates were incubated at 37 °C, 5% CO2, 95% humidity for 48 h. Following the infection, media was removed and TZM-bl cells were lysed using 1× lysis buffer (25 mM Gly-Gly pH 7.8, 15 mM MgSO4, 4 mM EGTA, 1% Triton X-100) containing Bright-Glo^TM^ luciferase substrate (Promega). Virus infection of TZM-bls was assessed by luciferase intensity read on a Biotek Synergy 2 (Biotek). Infectivity data was transformed into percent virus neutralization using the equation:

$$	 \% \,{neut}= \\ 	 100\times \frac{\left({RLU}\,{of}\,{sample}-{average}\,{RLU}\,{of}\,{cell}\,{control}\right)}{\left({average}\,{RLU}\,{of}\,{virus}\,{control}-{average}\,{RLU}\,{of}\,{cell}\,{control}\right)}$$

The serum dilution factor (SDF) values that resulted in 50% inhibition of viral infectivity (IC_50_) based on fitting of the % neut vs. log SDF data was calculated when possible. Functional VRC01 was quantified using the known VRC01 neutralizing IC50 for 001428_2_42 (0.019 µg/ml) which was confirmed using a VRC01-P2A-IgG standard.

### Total antigen-specific IgG response enzyme-linked immunosorbent assay (ELISA)

384-well ELISA plates (Corning, 3700) were coated with 12.4 μl/well of 2 µg/ml streptavidin (JacksonImmuno #016-000-084) in PBS and incubated at 4 °C overnight. Plates were washed 3x with 100 μl/well PBS containing 0.05% Tween (PBS-T) and blocked with 60 μl/well PBS  +  3% BSA at RT. After one hour, plates were washed and 12.4 μl of 2 μg/ml biotin-labeled AVI-tagged BG505 SOSIP was diluted in PBS-T and 1% BSA and added to each well. After washing (as above), rhesus sera were serially diluted (6x) using PBS-T and 1% BSA starting at 1:6 in 12.4 μl/well and incubated at RT for one hour. After washing, 12.4 μl/well alkaline phosphatase-conjugated goat anti-human IgG (H  +  L) (Jackson Immuno, 109-055-003) diluted 1:3000 in PBS-T, 1% BSA was added and incubated for 30 min at RT. Plates were washed and p-nitrophenyl phosphate (pNPP) substrate (Sigma Aldrich) dissolved to 1 mg/mL in substrate buffer (10 mM MgCl2 with 80 mM Na2CO3 and 15 mM NaN3, pH 9.8) was added at 12.4 μl/well to visualize the binding of antigen-specific mouse IgG. After 90 mins, optical density (OD) at 405 nm was read on an Epoch microplate spectrophotometer (Biotek). EC50 values were calculated by fitting curves to plots of Absorbance values vs. the log of the SDF for each sample using Graphpad. Variable slope (four parameter) functions with minimum (background) and maximum value constraints were used to fit curves using Prism 10 software (Graphpad). The same mass amount of biotinylated eOD-GT5 or ZM197-trimer were used instead of MD39-trimer to detect host responses to these antigens.

### Anti-P2A enzyme-linked immunosorbent assays

For total P2A titer, 384-well plates were pre-coated overnight at 4 °C with 12.4 μl/well, 2 μg/ml eOD-GT8-60mer [54] in PBS. Plates were washed 3x with 100 μl/well PBS containing 0.05% Tween (PBS-T) and blocked with 60 μl/well of PBS supplemented with 3% BSA at RT for 1 h and washed again. Rhesus serum samples serially diluted (4x) with PBS-T and 1% BSA starting at 1:4 dilution were added (12.4 μl/well) and incubated at room temperature for one hour. A P2A-LC tagged human VRC01 IgG monoclonal antibody standard starting at 1 μg/mL was also added (12.4 μl/well), serially diluted (4x), and incubated at RT for one hour. Plates were washed and incubated with 12.4 μl/well biotin-labeled anti-P2A-peptide mouse antibody (Millipore Sigma, MABS2005) at 1 μg/ml in PBS-T and 1% BSA. The anti-P2A antibodies were biotinylated in-house (Thermo Scientific, PI21450). Plates were washed and incubated with 12.4 μl/well alkaline phosphatase-conjugated Streptavidin (Jackson Immuno) at 1:3000 dilution in PBS-T and 1% BSA at room temperature for one hour. Plates were washed and pNPP substrate was added, developed and read at 405 nm as above. SDF EC50 values were calculated for serum samples by fitting a curve to Absorbance vs. log SDF data points from the titration and P2A-contaning antibody concentrations in the serum could be quantified by multiplying this number to the EC50 for the standard curve. The lower limit of detection (LOD) was 4 ng/µl which corresponds to the concentration that gives an Absorbance signal that is three-times background levels. For isotype-specific P2A-titer, 384 well plates were pre-coated overnight at 4 °C with 12.4 μl/well, 2 μg/ml anti-P2A antibody (Avidity LLC, BirA500). The following day, plates were washed, blocked, and serum added as described above. Isotype-specific alkaline phosphatase-conjugated antibodies (Jackson Immuno, anti-IgG: 109-055-003, anti-IgM: 50-194-3543) were diluted to 1:1000 (anti-IgG) or 1:3000 (anti-IgM) in PBS-T and 1% BSA and added at 12.4 μl/well. Plates were washed and pNPP substrate was added, developed for 90 min before being read at 405 nm as above. P2A-titers could be quantified for IgG using a P2A-tagged IgG standard as above. IgM titers were reported as SDF EC50s. Variable slope (four parameter) functions with minimum (background) and maximum value constraints were used to fit curves using Prism 10 software (Graphpad) for unknowns and the standard. EC50 in SDF factor for unknowns were multiplied by EC50 values in µg/ml for the standard to obtain EC50s for unknowns in µg/ml.

### Flow cytometry and sorting

Flow cytometry was used to detect engineered B cells using antigen-probes with specificity for reprogrammed BCRs, and for phenotyping cells cultured in activation media or SLOs as well as cells obtained from NHP PBMC or LN biopsy samples. Specific probes and protocols used for the various panels are presented in the table below. For analysis of cryopreserved samples, cells were thawed by quickly swirling cryotube in 37 °C water bath until just thawed and added to 10 ml of prewarmed supplemented IMDM media, spun at 800xg for 10 min, washed a second time with media, and then counted. Cells were then spun down in 5 ml polystyrene tubes (Falcon) and washed with PBS + 2% FBS. For live/dead staining, 1×10^6^ cells were resuspended in 1 ml 1:1000 live/dead stain in PBS and incubated in the dark at RT for 30 mins. Cells were then washed with PBS + 2% FBS and spun again. For surface staining, 1×10^6^ cells were resuspended per 100 µl of stain and incubated in the dark at RT for 20-30 mins (for engineered cell detection panel) or 45 mins (cell surface B cell phenotyping panel), then washed with PBS + 2% FBS and spun again. To prepare probes for detection of reprogrammed cells, biotinylated anti-P2A antibody (random biotinylation; Thermo Scientific, 21450), eOD-GT8/KO11, or native trimer probes (Q23, MD39) were conjugated with streptavidin fluorophores at appropriate molar ratios (4-Env-trimers: 1-strep-tetramer) and incubated in the dark for 15 mins before use. For intracellular staining, either True-Nuclear Transcription Factor Buffer Set (Biolegend, 424401) or Foxp3 Fix/Perm Solution (Invitrogen, 00-5523-00) were used according to manufacturer instructions. If intracellular staining was not required, cells were spun down after surface staining and resuspended in 100 µl PBS + 2% PFA and incubated at RT for 15 min, then washed and resuspended for analysis. Stained cells were resuspended in PBS + 2% FBS and acquired on a 5 L Cytek Aurora. Data was analyzed using Cytobank or FlowJo. For FACS, cells were washed once in PBS + 2% FBS then stained with probes/antibodies in PBS for 30 min at RT, if applicable. Cells were washed once in 2 PBS + 2% FBS, stained with antibodies for 45 min at 4 °C, washed twice in PBS + 2% FBS, and then resuspended in RPMI without phenol red. FACS was done on a BD AriaFusion.

**Table:** 

Antibody	Fluorophore	Cat No	Dilution factor or (µl/100ul stain)
Live/dead	Blue	Thermo Fisher L34961	1:1000 (stained separately)
FcX	NA	Biolegend 422301	10
BV Buffer	NA	BD 566385	10
CD3	BUV805	BD 742053	5
CD14	BUV805	BD 612903	2.5
CD20	BV605	Biolegend 302333	2.5
IgD	AF488	Southern Biotech 2030-30	1.25
CD27	BV711	Biolegend 356429	10
CD21	BUV737	BD 612789	5
CD11c	APC-R700	BD 566610	2.5
CD38	PerCP-Cy5.5	Caprico 100864	0.6
Lambda	APC-vio770	Miltenyi 130-122-223	1
Bcl-6	BUV496	BD 567753	5
Ki67	BV786	BD 563756	1.25
CD80	PE	BD 557227	5
Biotinylated-KO11	BV421	Provided by Schief Lab (Scripps)	1
Biotinylated-eOD-GT8	AF555	Provided by Schief Lab (Scripps)	1
Biotinylated-eOD-GT8	AF647	Provided by Schief Lab (Scripps)	1
Biotinylated-MD39	AF555	Provided by Schief Lab (Scripps)	2
Biotinylated-MD39	AF647	Provided by Schief Lab (Scripps)	2
Biotinylated- Anti-P2A	AF647	Millipore Sigma, MABS2005	1
Biotinylated- Anti-P2A	AF555	Millipore Sigma, MABS2005	1

### VRC01 cDNA amplification and gel

B cells were enriched from lymphocytes by MACS or specific populations were sorted by FACS. For bulk RNA libraries from sorted PBMCs, the following populations were sorted: Live CD20^-^IgD^-^CD27^-^CD80^+^CD38^+^, Live CD20^+^IgD^-^CD27^+^, LiveCD20^+^IgD^+^CD27^-^, and Live CD20^-^IgD^-^CD27^-^CD80^-^CD38^+^. Cells were sorted into RPMI with 5% FBS, centrifuged at 500xg for 10 min, then resuspended in 350 µl RLT/BME, vortexed for 1 min, and put at -80C. For bulk RNA libraries from B cells isolated from PBMCs or lymph node biopsies, B cells were isolated as described above. mRNA was purified (Qiagen), which was immediately used to generate cDNA using first-strand synthesis and random hexamers (Invitrogen) according to manufacturer instructions. Resulting cDNA was used as template in nested PCR reactions using HF-Phusion (Thermo Scientific, F530L). Primers were annealed for 30 sec at 53 °C for PCR1 and 52 °C for PCR2, with a subsequent extension time of 30 s at 72 °C. PCR products with 1X orange G were loaded onto a 1% agarose gel with 1X SybrSafe (Invitrogen) and run at 100 V in 1X TAE buffer, then imaged on a ProteinSimple gel imager.

**Table:** 

PCR1	14 cycles
Forward 1	TTCTGAAACAGGCCGGAGA
IgM reverse 1	TACTTGCCCCCTCTCAG
IgG reverse 1	TTGTCCACCTTGGTGTTG
PCR2	34 cycles
Forward 2	TGGAGGAGAATCCTGGA
IgM reverse 2	CATTCTCACAGGAGACGAG
IgG reverse 2	CCCTGAGGACTGTAGGA

### Recombinant antibody standards

BnAb VJ-P2A-VDJ constructs (with mouse or macaque kappa constant genes) were PCR amplified from pITR plasmids and cloned into a mouse (pFUSE-CHIg-mG1, Invivogen) or NHP IgG expression vector (pFUSE-CHIg-rhG1, Invivogen) using Gibson assembly. For mouse anti-P2A antibodies, a P2A encoding LC and HC were cloned into mouse vectors AbVec2.0-mIgkc and AbVec2.0-mIghg1, respectively (Addgene 127157, 127158). Purified (Qiagen) sequence confirmed (Plasmidsaurus) expression vectors were transfected into Expi293 cells using FectoPRO (Polyplus, 101000007) and Opti-MEM to achieve transient expression of the antibody. Expi293 cells were adjusted to a density of 2.8×10^6^ cells/ml in 108mls of Expi293 Expression Medium. 96 µg of transfection plasmid and 96 µl of FectoPRO were mixed in 12mls total volume with pre-warmed Opti-MEM and incubated at RT for 10 min before adding to prepared Expi293 cells. Cells were fed 24 h after transfection with 1.2 mL (1% culture volume) each of 45% D-glucose and 300 mM valproic acid. Supernatants were harvested 5 days post transfection, and IgG was affinity purified using Protein G Sepharose resin (Cytiva, 17061805). Supernatants were filtered through 0.22 µm PES membranes and incubated overnight at 4 °C, rotating with 1 ml Protein G resin. The following day, supernatants were added to chromatography columns for gravity-flow purification. Columns were washed with ten column volumes of PBS. IgG was eluted with 0.2 M citric acid pH 3.0 into 20% elution volume 2 M Tris Base pH 9.0, then immediately buffer exchanged into PBS with 50 K MWCO Amicon tubes (Millipore Sigma, UFC905008).

### Surface plasmon resonance

Kinetics of antibody-antigen interactions were measured on a Biacore 8 K (Cytiva). Experiments were performed at 25 °C at a flow rate of 30 µL/min in a mobile phase of HBS-EP+ (Teknova). Anti-human Fc IgG antibody was immobilized on a CM3 chip (Cytiva, BR100541) using the Human Antibody Capture Kit (Cytiva, BR100839) according to manufacturer instructions. bnAbs expressed as rhesus IgG were injected over the immobilized CM3 chip, followed by a wait period for normalization of response units (RU). Regeneration was accomplished using 3 M MgCl_2_ with 30 s contact time. A concentration of MD39-trimer ranging from 15.625–500 nM was injected across the antibody and control surface for 120 s, followed by a 500 s dissociation phase in PBS buffer alone. MD39-trimer binding on (k_on_) and off (k_off_) rates were assessed and KD were calculated using BIAEvaluation software (Cytiva).

### Single Cell RNA sequencing and analysis of culture B cells

Rhesus macaque single-cell cDNA libraries were prepared using the 10x Genomics Chromium Next GEM Single Cell 5’ Kit (V2 chemistry) and the 10x Genomics Chromium Next GEM-X Single Cell 5’ Kit (V3 chemistry). The cDNA libraries were used to generate and GEX libraries according to the manufacturer instructions. The libraries were quantified using Agilent Tapestation and sequenced with Illumina NextSeq 1000/2000 kits. Fastq files were generated from raw sequencing base call (BCL) data using CellRanger (8.0.1) mkfastq. The fastqs from the 5’ GEX libraries generated from the cell culture samples were processed using Cellranger count with a custom Rhesus macaque reference. The reference was sourced from Ensembl and updated to include VRC01 HC and LC IGV-gene sequences, and unannotated genes were removed. Ig constant genes that were unannotated in the reference (IgD, IgA, and IgG1) were annotated using pantherdb [82]. Scanpy [83] was used to filter cells out if they expressed fewer than 100 genes and genes were discarded if they were present in fewer than three cells. Mitochondrial genes were assessed to determine quality of cells and dataset [84]. A principal component analysis was executed to reduce the dimensionality of the gene counts matrix, then the cells were Leiden clustered [85] and neighbors were embedded using UMAP [86]. Ig variable genes were removed from the dataset prior to clustering and embedding. Engineered cells were identified as positive for VRC01 IGV-gene expression. Endogenous IGHV-gene transcription could be assessed in subsets of unengineered cells when they expressed IGHV-genes that could align with greater than 90% identity (Scanpy) to several annotated IGHV-genes in the reference sequence. Isotype was determined based on raw expression values of *IgH* constant genes reads above an expression threshold of 250 reads/cell.

### Organoid culture and vaccination

Organoids were cultured as previously described [70, 71]. Cryopreserved spleen or LN biopsied cells were cultured in 96-well transwell plates (Corning, 07200278) in RPMI with Glutamax (Gibco, 35050061), 10% FBS, 1× nonessential amino acids, 1× sodium pyruvate, 1× penicillin–streptomycin, 1× Normocin (InvivoGen, ant-nr-2) and 1× insulin/selenium/transferrin cocktail (Gibco), 1 μg/ml of recombinant human BAFF (BioLegend, 559602) at 1.5 × 106 cells/well. 30% of media was changed from the base well every other day beginning on day 1 of culture (24 h after cultures began). For vaccinations, Adju-Phos (InvivoGen, vac-phos-250) and Alhydrogel (InvivoGen, vac-alu-250) were combined with immunogen according to manufacturer instructions. For imiquimod (InvivoGen, vac-imq) and SMNP, 0.05 µg per transwell were used for each vaccination. Vaccinations were added directly to the transwell on the first day of culture. Engineered cells were vaccinated with 1 µg/ml MD39-ferritin and SMNP. Samples were assessed by flow cytometry using B cell phenotyping panel described above. Cells were removed from transwells by pipetting up and down several times, and transwells were washed with PBS with 2% FBS to remove residual cells before spinning down and proceeding to flow cytometry staining.

## Supplementary information

supplementary figures

## Data Availability

NGS data available on NCBI SRA (PRJNA1402441) and metadata is available on NCBI GEO (GSE317633). Script required for custom analysis of 10X genomics data available at github.com/jamesevoss/NHP_B_cells. Donor DNA designs for VRC01 (PQ369283), Ab1485 (PQ369284), RHA1.V2.01 (PQ369285), RHA1.V2.01 HC only (PQ369286), sVRC01 1138 (PV862354), and sVRC01 16001 (PV862355) have been deposited in Genbank. These HRTs are available as ITR plasmids from Addgene (251808, 251809, 251810, 251811, 251812, 251813).
