# Supplementary material for: Vaccine elicitation of HIV broadly neutralizing antibodies from genome-edited B cells in non-human primates and derived lymphoid organoids
Source: Gene Ther. 2026 Apr 10;33(5):541–58. doi: 10.1038/s41434-026-00610-8 (PMC13585563; doi:10.1038/s41434-026-00610-8)
Supplement: Supplementary file 1 [file 41434_2026_610_MOESM1_ESM.pdf]

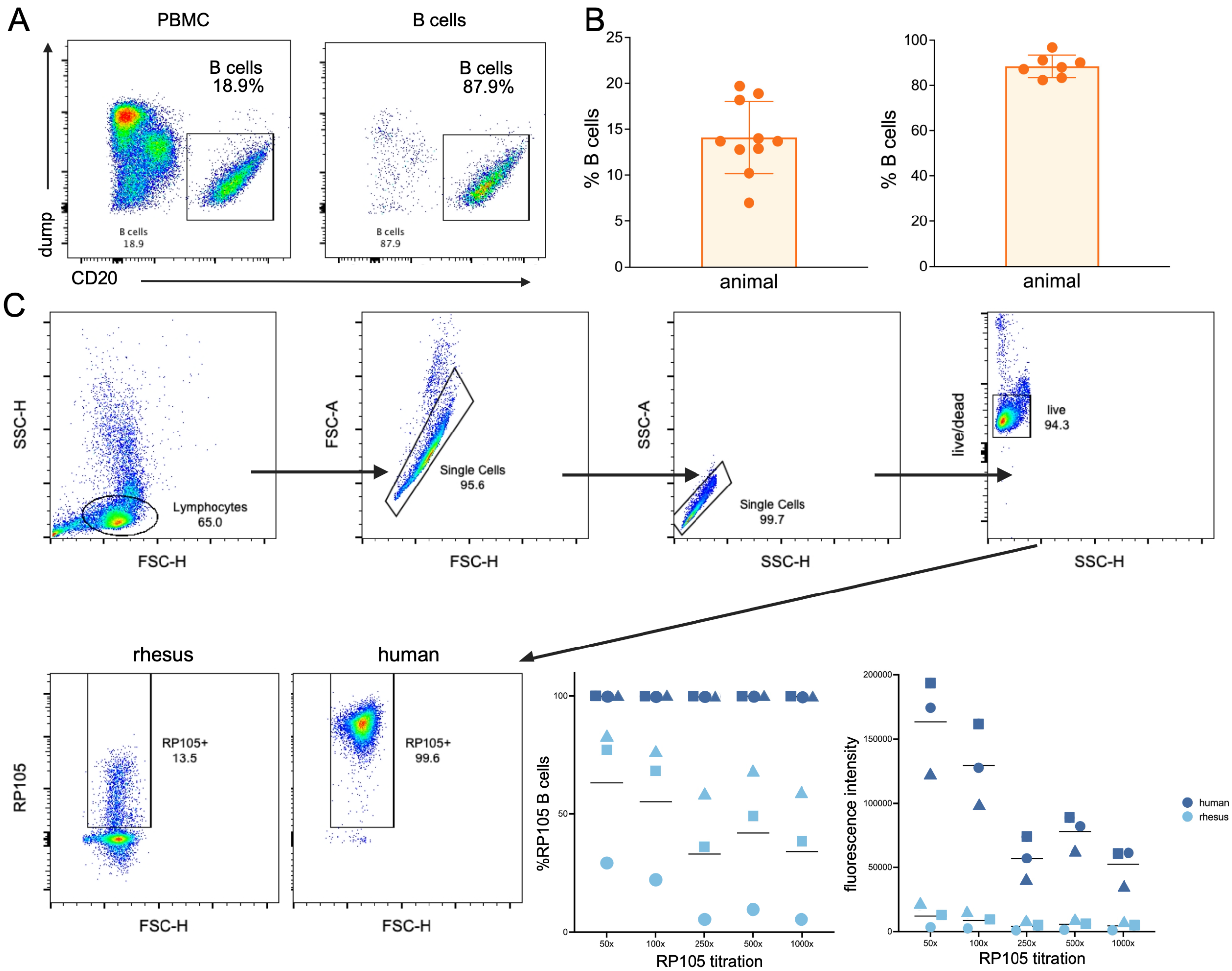

Figure S1. B cell purification and activation optimization. A) Representative sample of B cell isolation results from frozen PBMCs showing B cell frequency in the starting PBMC population (left) and B cell purity post isolation (right). B cells were gated as CD20^{+}, dump (CD3, CD4, CD8, CD14)$^{-}$ live lymphocytes. **B)** Starting B cell frequency in PBMCs (n=10) (left) and resulting B cell purity after B cell isolation using negative selection (n=7) (right). **C)** Flow cytometry gating strategy to assess RP105 binding of rhesus and human B cells (top, bottom left). Example of RP105 binding populations at 100X RP105 staining dilution (bottom left). RP105 expression comparison between human and rhesus macaque B cells (n=3 donors each) where frequency of RP105^{+} B cells and surface expression level (mean fluorescence intensity of RP105 stain) are shown (bottom right).

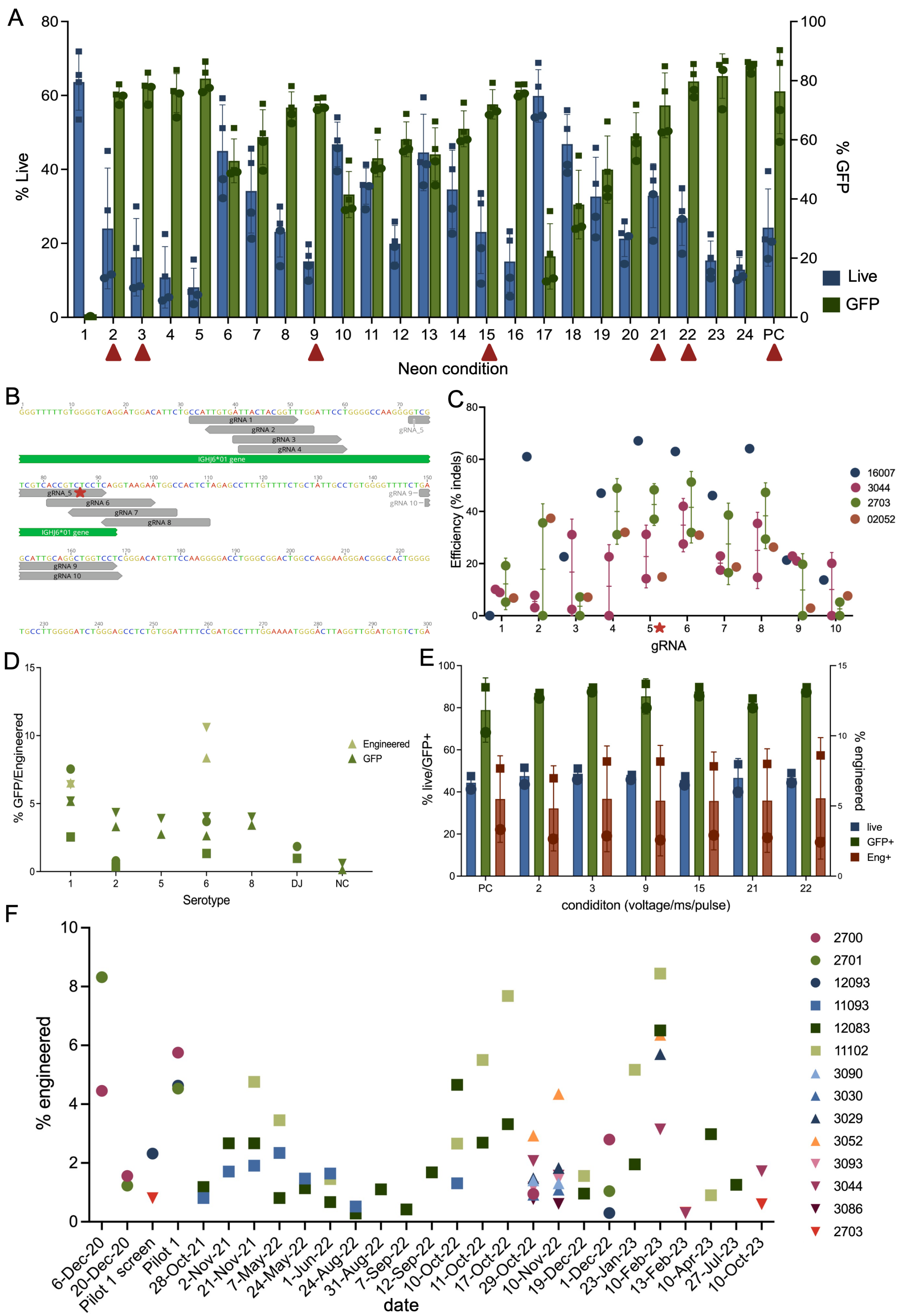
A
% Live
% GFP
Live
GFP
Neon condition
B
C
Efficiency (% indels)
gRNA
16007
3044
2703
02052
D
% GFP/Engineered
Engineered
GFP
Serotype
E
% live/GFP+
% engineered
live
GFP+
Eng+
condiditon (voltage/ms/pulse)
F
% engineered
date

Figure S2. Engineering optimization. A) Optimization of NEON electroporation conditions using GFP mRNA, where B cells were activated using CD40L, IL-2, IL-4, and CpG media and electroporated using the Neon 24-well suspension cell buffer R optimization protocol. N=2 donors (circle = donor 1, square = donor 2), and the experiment was performed in duplicate. Red triangles indicate nucleofection conditions yielding higher cell viability and GFP signal (>15% and 70%, respectively). **B)** 10 gRNAs were designed to a 300bp region downstream from the IGHJ6-gene using Horizon Discovery as indicated in the linear diagram. Forward or reverse compliment gRNAs in grey are indicated by the arrow direction. **C)** Ability of various gRNAs to generate INDELs at target sites in NHP B cells from several indicated donors was assessed by TIDE. Percentage of target sites with INDELS for each sample 48 hrs after RNP nucleofection are shown. **D)** GFP-reporter AAVs as various serotypes were tested for their ability to transduce activated NHP B cells. Percent GFP positive cells were measured by flow cytometry after a 24hr transduction (dark green). Each symbol represents a different animal. We tested engineering with VRC01 donor DNA packaged as AAV1 or AAV6 (light green). AAV1 was tested as this serotype results in the highest transduction efficiency and AAV6 was selected as this serotype had previously been used with human B cells. AAV6 engineering efficiencies trended higher. **E)** Best electroporation conditions (red triangles in A) were used in combination with VRC01 HRT AAV6 transduction and engineering was assessed 48 hours later using flow cytometry to detect VRC01 antigen binding (orange). Cell viability (blue) and expression of nucleofected GFP mRNA (green) for each condition are also shown N=2 donors (circle = donor 1, square = donor 2). **F)** Engineering efficiencies over time across several animals and samples, showing that efficiency ranges from between approximately 1-9%.

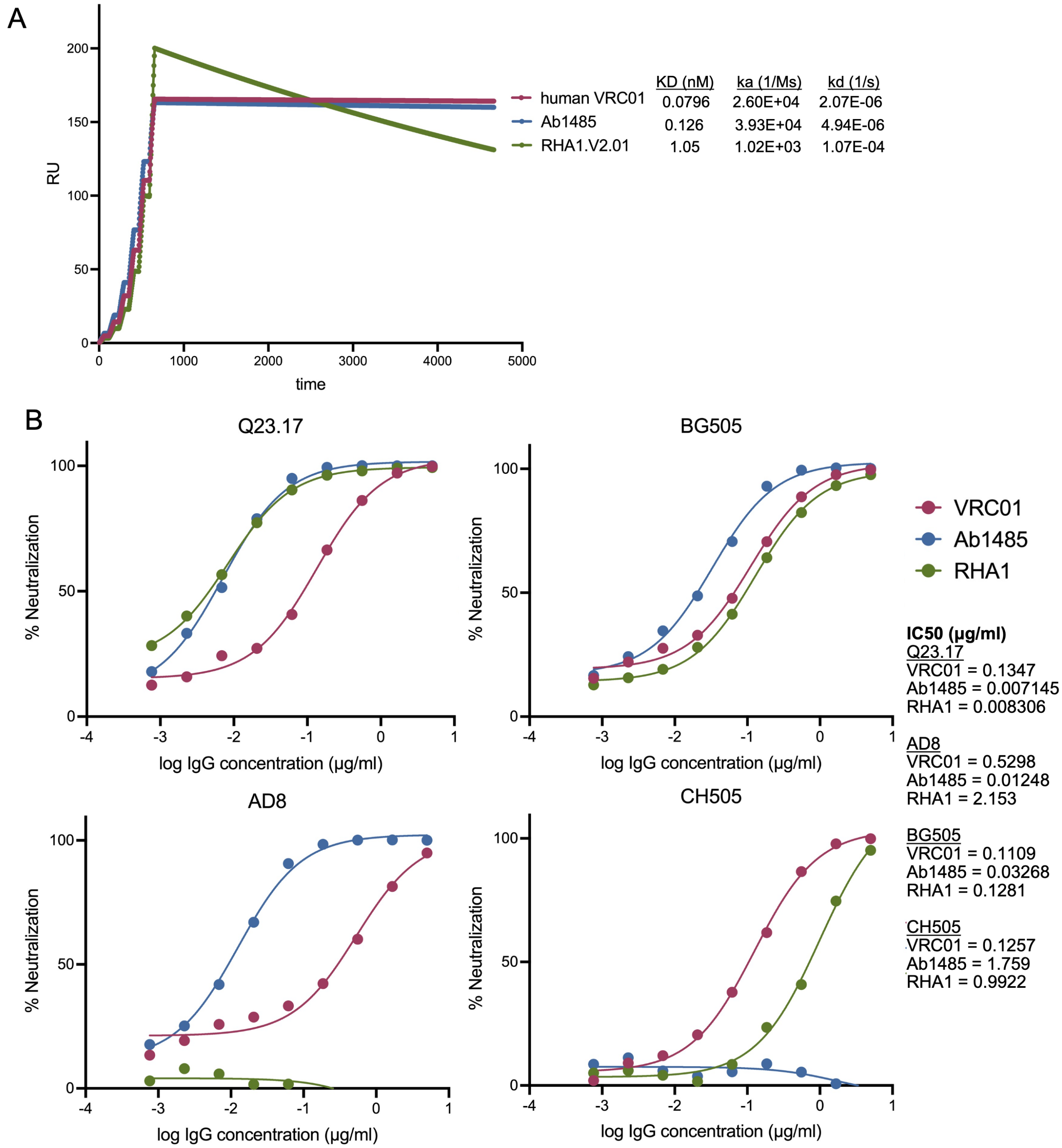

Figure S3. Extended bnAb data. A) Bivalent KDs of authentic rhesus macaque bnAbs and human VRC01 with MD39 trimer. **B)** Percent neutralization of the indicated HIV psuedovirus is plotted as a function of the log of the bnAb concentration for VRC01, Ab1485 and RHA1.v2.01. IC50 values are indicated.

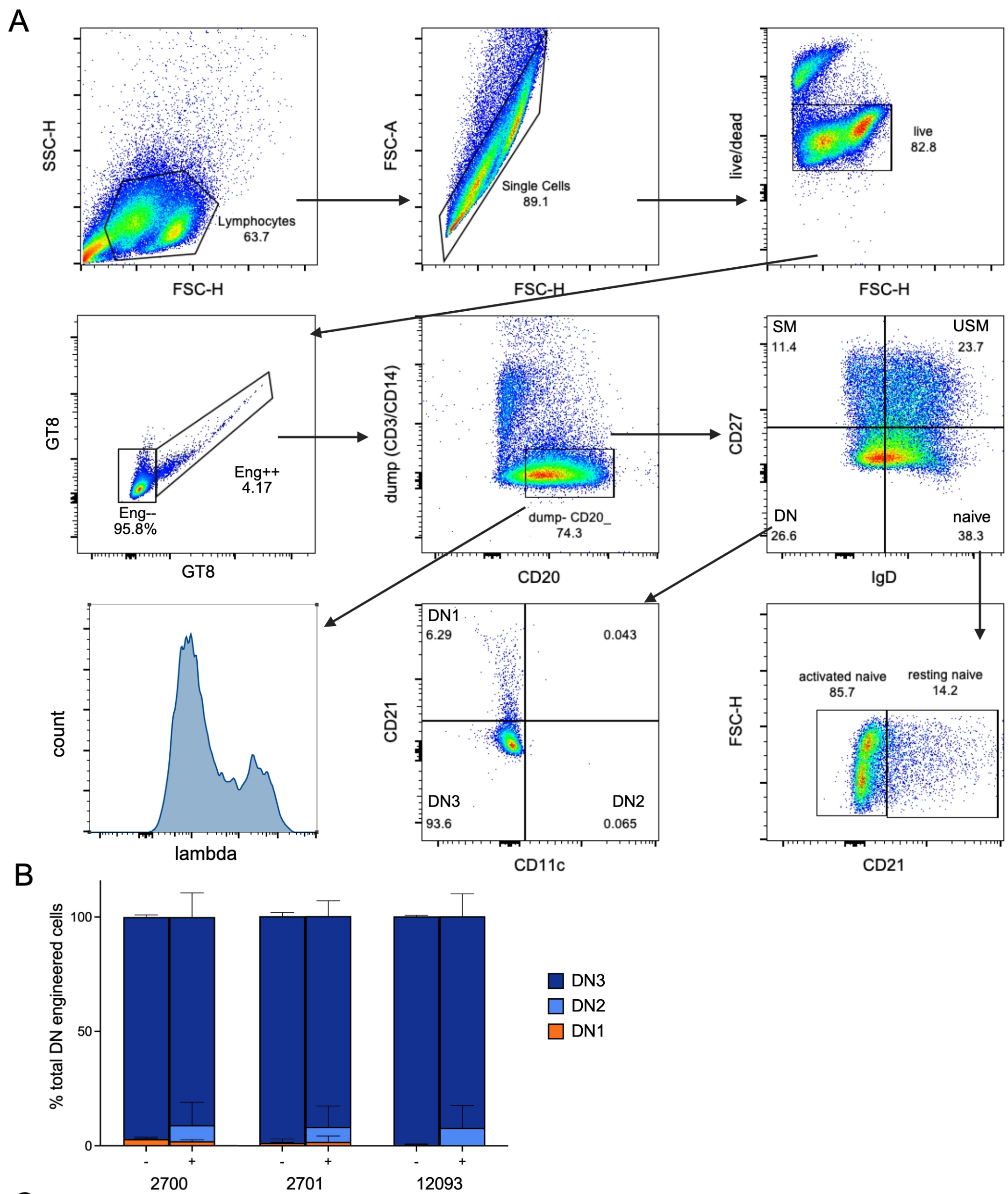

C

cluster	VRC01-					VRC01+					% VRC01+
	total	IgM	IgG	IgA	IgE	total	IgM	IgG	IgA	IgE	
0	285	0	0	0	0	160	0	0	0	0	36
1	101	0	0	0	0	339	0	0	0	2	77
2	299	0	0	0	0	22	0	0	0	0	6.9
3	43	4	10	2	0	245	41	41	5	0	85
4	243	1	0	0	0	12	0	0	0	0	4.7
5	24	0	4	4	0	215	17	40	3	6	90
6	92	0	0	0	0	116	0	0	0	1	56
7	117	0	0	0	0	14	0	0	0	0	11
8	67	0	1	0	0	14	1	2	0	0	17

Figure S4. Phenotype and allelic inclusion of engineered cells. A) Representative gating strategy for B cell phenotyping. This figure shows targeted B cells on day 6 of culture in pilot-1 activation media. Engineered B cells are first gated from live, single lymphocytes using eOD-GT8 antigen. Engineered cells

are defined as KO11$^-$ GT8^{++} where KO11 is GT8 with point mutations that knock out VRC01 binding to its epitope. Engineered or unengineered populations can then be assessed for CD27 and IgD expression to evaluate the frequency of USM (CD27$^+$, IgD$^+$), SM (CD27$^+$, IgD$^-$), naïve (CD27$^-$, IgD$^+$), and DN (CD27$^-$, IgD$^-$) B cells. DNs can be further phenotyped as DN1 (CD21$^+$, CD11c$^-$), DN2 (CD21$^-$, CD11c$^+$), and DN3 (CD21$^-$, CD11c$^-$) populations. Engineered and unengineered cells can also be stained for lambda LC expression frequency. **B)** Frequency of DN subsets for engineered (+) and unengineered (-) cells from three donor animals on day 6 of culture in Pilot-1 media is given as a percentage of total DN's for the samples shown in Figure 2C. **C)** Table of the total number of engineered (VRC01+) and unengineered (VRC01-) cells, as well as the number of cells with a clearly identifiable isotype, in each cluster determined by scRNAseq at the time of engraftment. Engineered cells account for 35% of total cells (FACS enrichment of VRC01 cells was done prior to sequencing). Isotype was determined based on raw expression values of *IgH* constant genes reads above an expression threshold of 250 reads/cell.

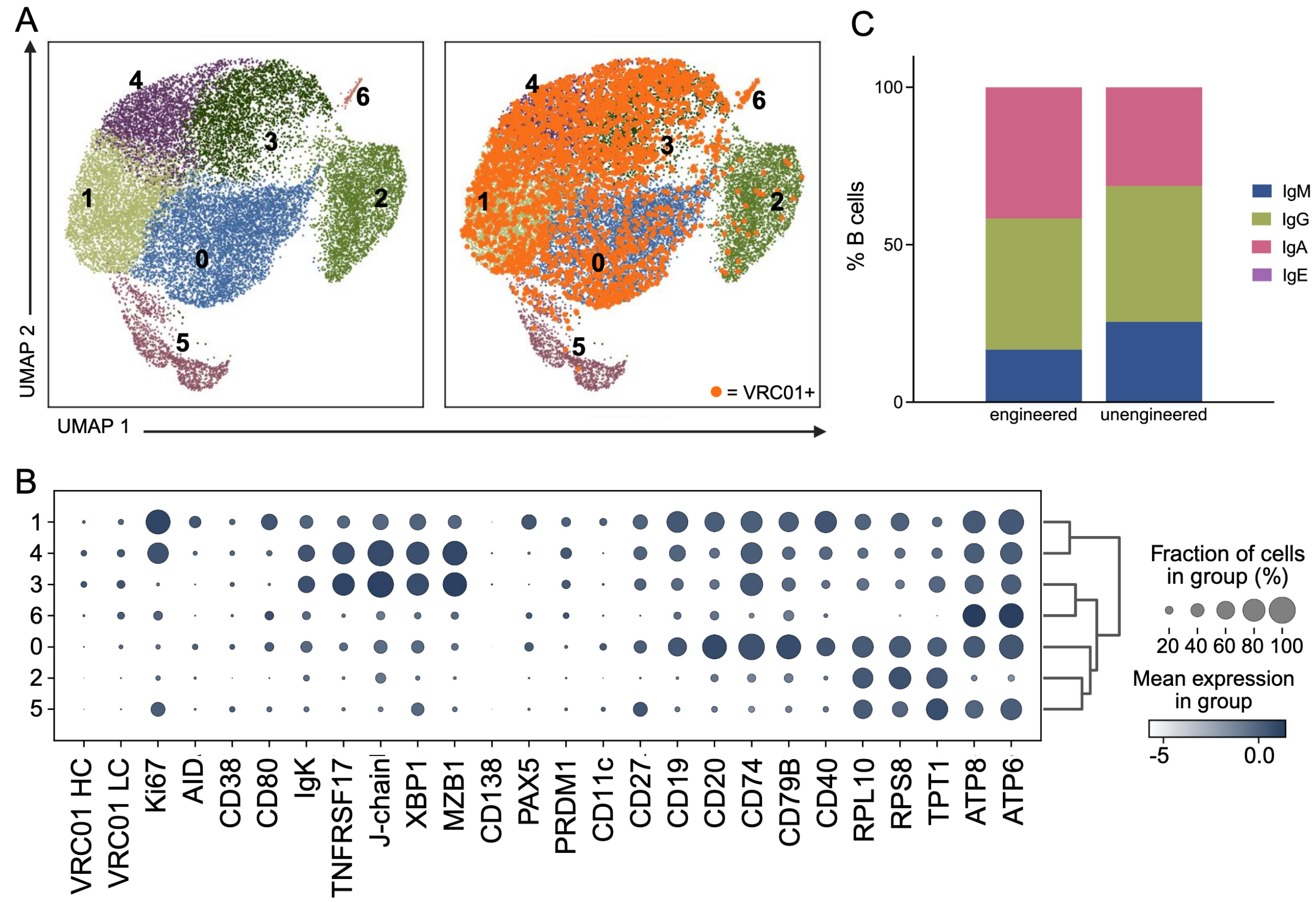

D

cluster	VRC01- total	VRC01- IgM	VRC01- IgG	VRC01- IgA	VRC01- IgE	VRC01+ total	VRC01+ IgM	VRC01+ IgG	VRC01+ IgA	VRC01+ IgE	% VRC01+
0	5703	0	0	0	0	561	0	0	0	0	9.0
1	3977	7	7	5	0	589	0	3	2	0	13
2	3475	0	0	0	0	65	0	0	0	0	1.8
3	2567	1	4	3	0	688	3	3	3	0	21
4	2320	4	10	8	0	560	2	4	4	0	19
5	1197	1	1	0	0	27	0	0	0	0	2.2
6	89	0	0	0	0	19	0	0	0	0	18

Figure S5. Single cell RNA transcriptomics characterization of *IgH*-reprogrammed B cells at the time of engraftment. A) UMAP Leiden clusters from scRNA-seq of an engineered pool of cells from a second animal at the time of engraftment is shown on the left. Cells with VRC01 transcripts are indicated in orange in these plots on the right. **B)** Dotplot showing transcriptional levels of VRC01 and other specific genes of interest are shown across all clusters. Relatedness of each cluster is shown. VRC01 cells are present in all clusters, but enriched mostly in clusters 0, 1, 3, and 4. Clusters 0 and 1 are defined by expression of canonical B cell markers. Increased Ki67 and CD80 expression in cluster 1 suggests this cluster is in cycle, likely toward a memory phenotype, while cluster 0 represents naïve B cells. Clusters 3 and 4 show lower transcription levels of canonical B cells markers and increased expression of VRC01, IgK, and ASC-associated genes. Cluster 4 maintains high levels of Ki67 expression, suggesting this cluster contains PBs, whereas cluster 3 is likely PCs. Clusters 2, 5, and 6 are defined almost exclusively by ribonucleoprotein and cellular respiration genes, which we believe to include our DN population by flow cytometry. The number of cells in these clusters aligns with the expected phenotype frequencies as determined by flow cytometry (Fig. 2A). **C)** Comparison of isotype frequencies of engineered cells compared to unengineered cells. *IgH* transcription levels were high enough only in ASC clusters 3 and 4 to determine isotype. Due to lower sequencing depth compared to the sample in Fig. 6, isotype was determined in a fewer number of cells. **D)** Table of the total number of cells, as well as number of cells per isotype, in each cluster for

engineered (VRC01+) and unengineered (VRC01-) cells determined by 10X single cell transcriptomics at the time of engraftment. Additionally, the percent of engineered cells in each cluster is shown. Isotype was determined based on raw expression values of *IgH* constant genes above an expression threshold of 250.

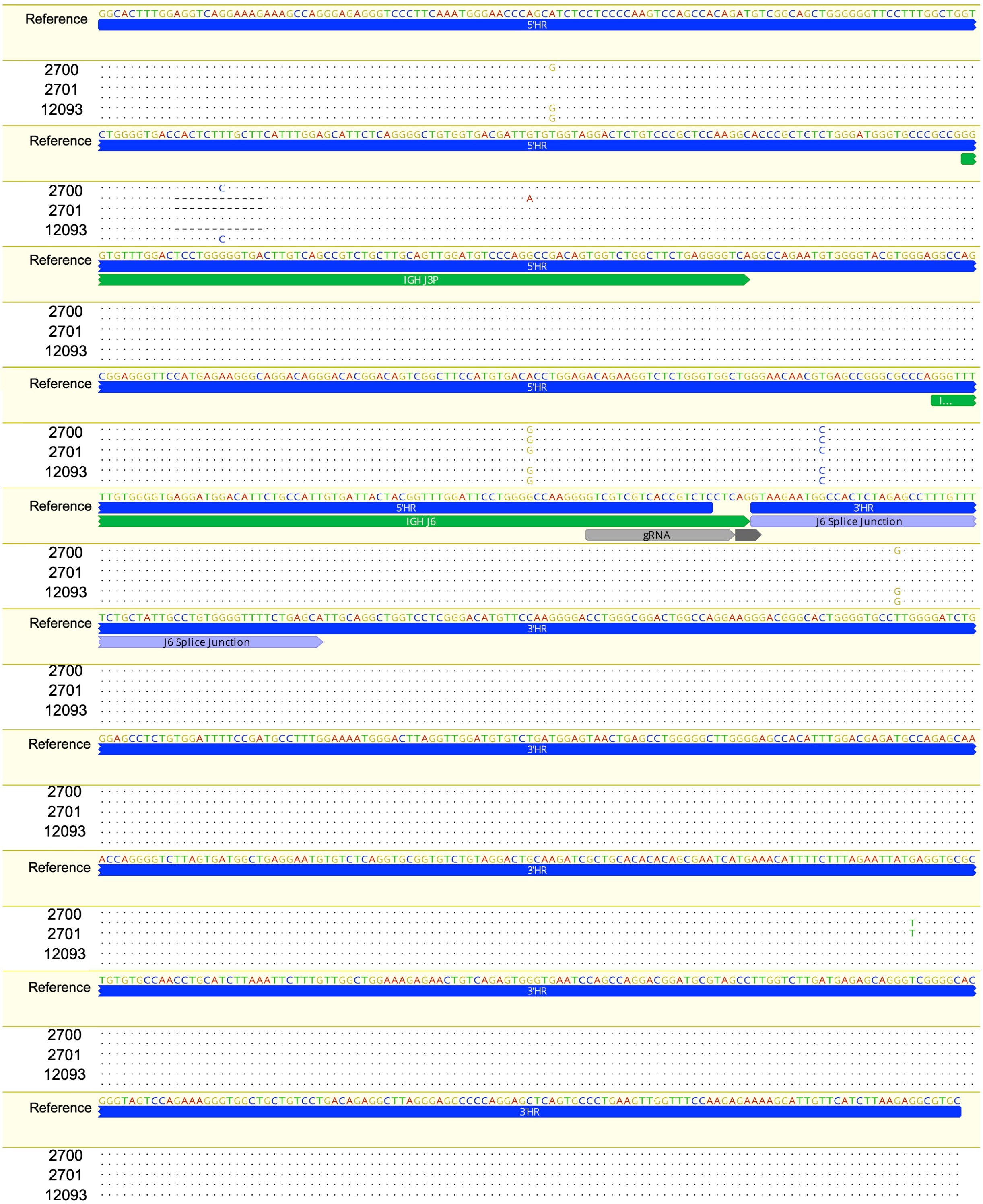

Figure S6. Pilot 1 donor homology arm alleles. Homology arms and gRNA target region were amplified from genomic DNA from PBMCs from animals selected for pilot experiments. These amplicons were topo-cloned and 5-10 cloned plasmids were sequenced to obtain sequences for both alleles. The gRNA and PAM site (dark grey) are intact for both alleles for all donors. Minor variations in the HA regions relative to the reference sequence are observed.

A

Animal	Sex	Age	Weight (kg)	MHC Haplotypes	Genotype	Engineering efficiency	Engineered cells transferred	Total B cells transferred
**r12093**	M	8.0	10.8	A019.01, A001.01, B001.01, B015.02, DR01.01, DR03.01, DQA_26g2, DQA_26_01, DQB_27g, DQB_18g3, DPA_02g1, DPA_02g3, DPB_15g, DPB_07g1	TFP/Q	4.7%	$1.0x10^6$	$2.1x10^7$
**rh2700**	M	7.6	9.2	A002.01, A008.01, B012.01, B012.01, DR03.01, DR01.01, DQA_26_01, DQA_26g2, DQB_18g3, DQB_27g, DPA_02g1, DPA_02g1, DPB_05_01, DPB_05_01	TFP/Q	4.0%	$1.2x10^6$	$3.0x10^7$
**rh2701**	M	8.6	12.1	A004.01, A008.01, B012.02, B069.01, DR04.01, DR04.01, DQA_01g1, DQA_01g1, DQB_06_01, DQB_06_01, DPA_02g1, DPA_02g1, DPB_15g, DPB_15g	TFP/Q	4.2%	$0.5x10^6$	$1.2x10^7$

B

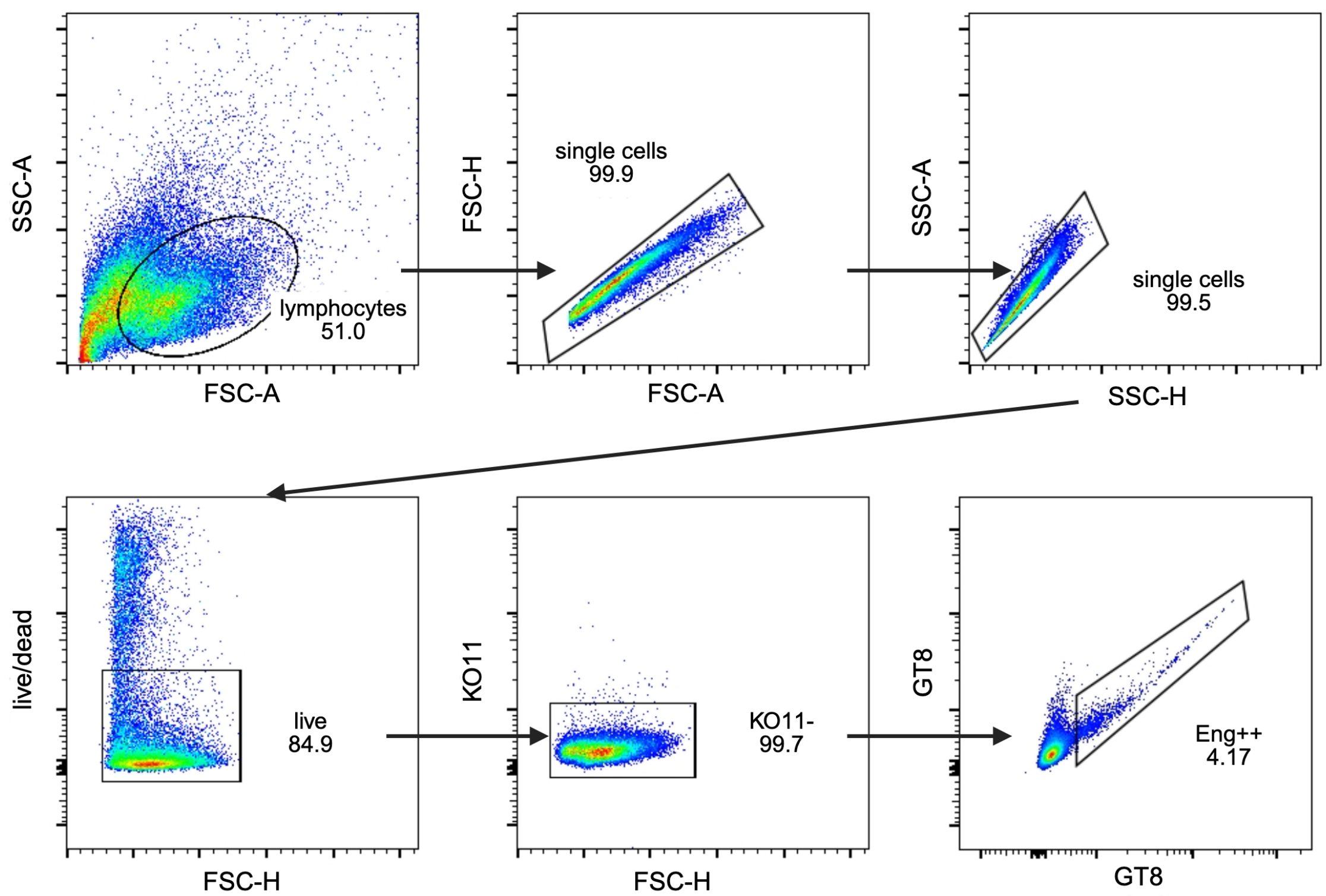

C

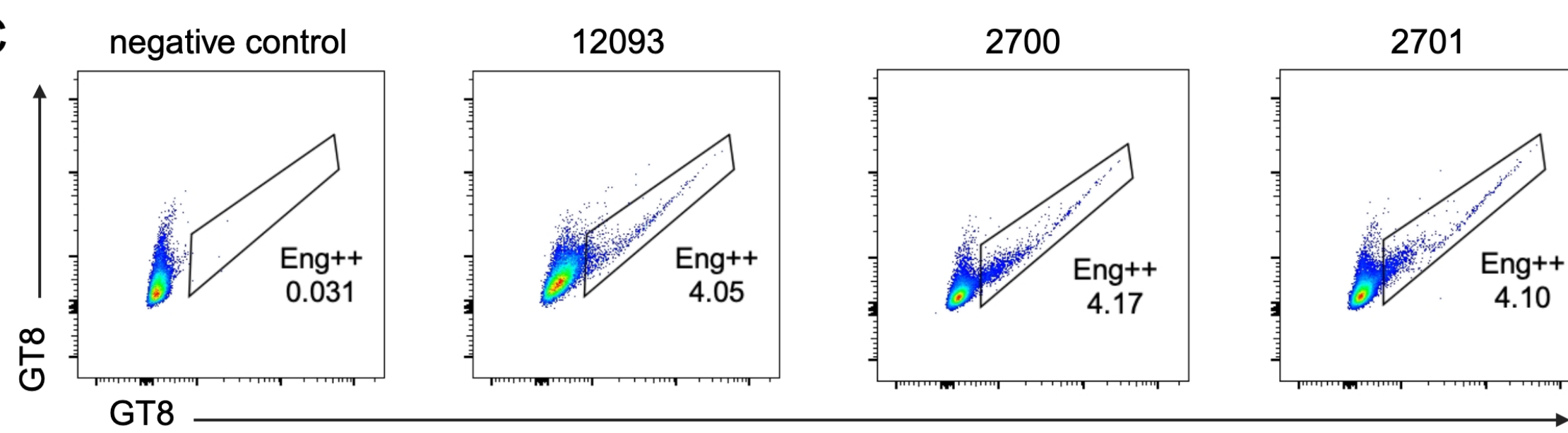

Supplementary Fig. 7 Extended pilot-1 setup data. A) Data table with information about pilot-1 animals and engineered cells generated and autologously engrafted. **B)** Example gating strategy to determine engineering efficiency for pilot-1 experiments using KO11- GT8++ gates. **C)** Engineering efficiencies for pilot-1 transfer animals compared to a representative unengineered negative control.

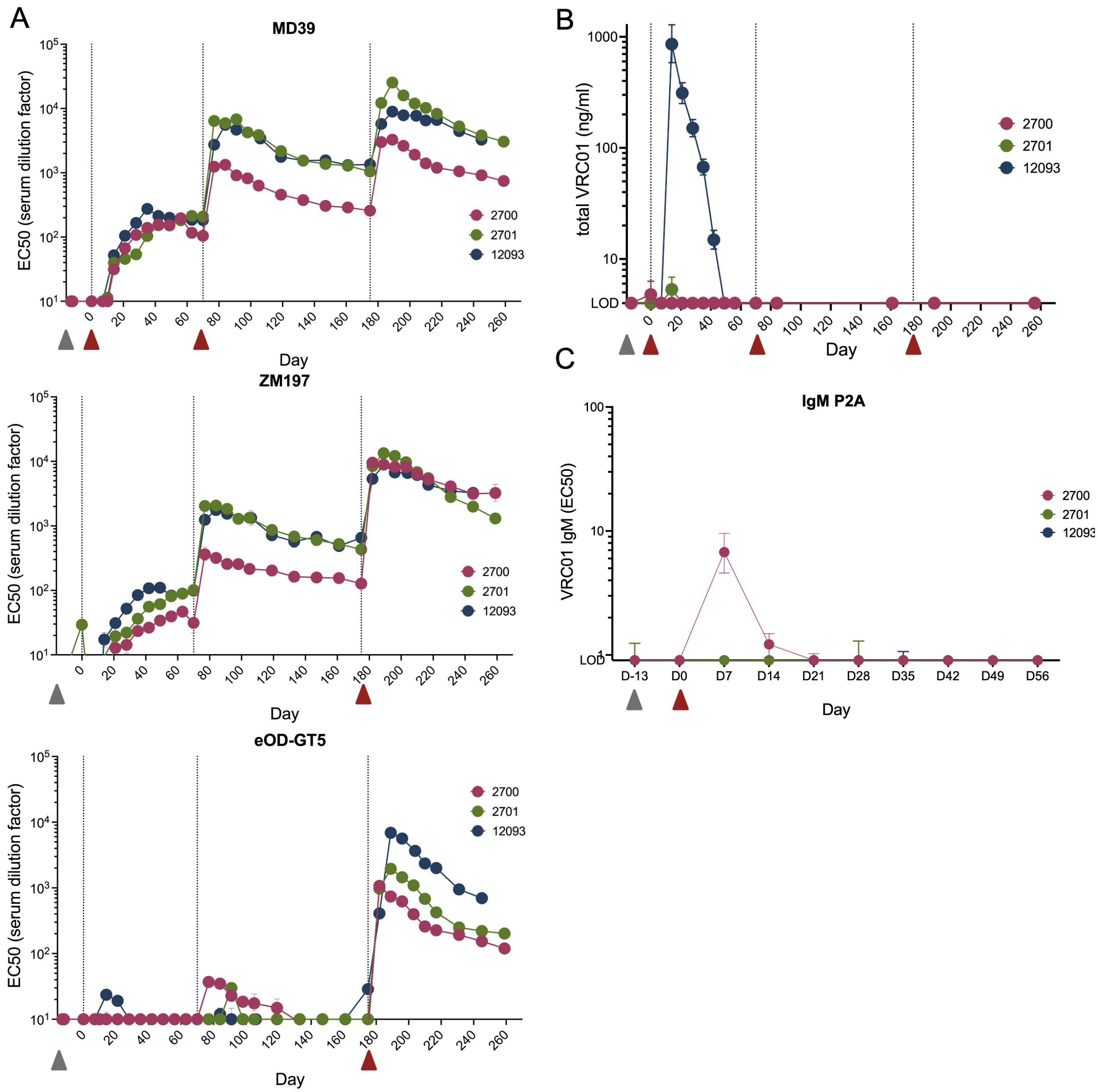

Figure S8. Pilot-1 extended serum responses. A) Total MD39-trimer (top), ZM197-trimer (middle) and eOD-GT5 (bottom) specific serum IgG titers in pilot-1 animals were measured over the course of the experiment and are given as SDF EC50s. MD39 titers were boosted by the heterologous final boost (ZM197-4mer and eOD-GT5-60mer). MD39-ferritin elicited antibodies that were cross reactive with ZM197-trimer. The grey triangle indicates engineered cell transfer and red triangles indicate the time of homologous immunization (i.e. immunization with an immunogen that is homologous to the probe being used in the ELISA). **B)** Extended total VRC01 serum antibody titers, as determined by anti-P2A ELISA including timepoints after the heterologous boost. Titers are given as antibody concentrations in ng/ml, which could be determined using a recombinant VRC01-P2A-IgG standard. **C)** IgM-specific VRC01 antibody titers in serum of animals after prime and boost, as determined by an isotype specific anti-P2A ELISA. Titers are given as SDF EC50s.

A

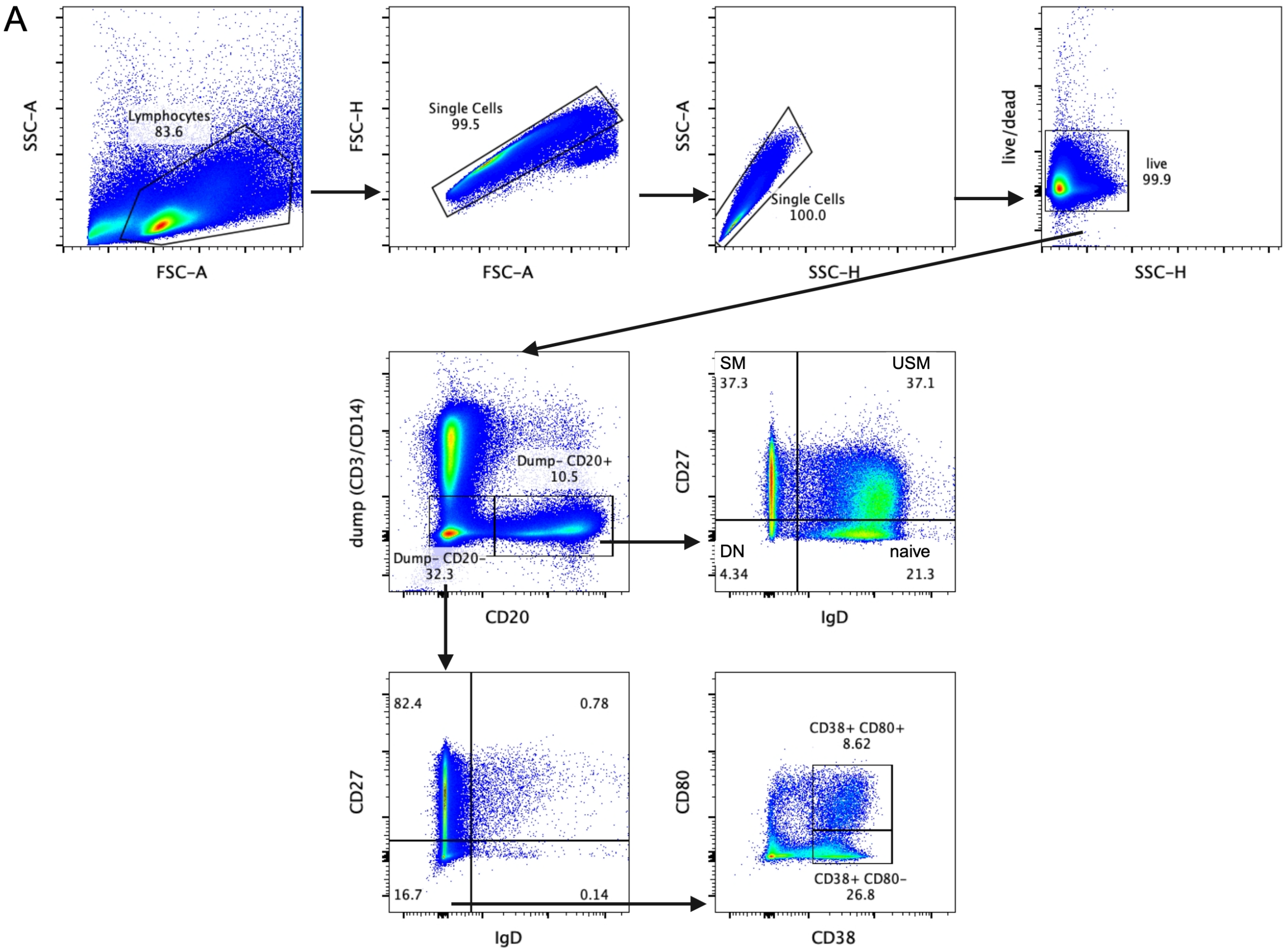

B

population	markers	phenotype	sort count
**1**	CD38+ CD80-/low	ASC like	4,426
**2**	IgD+ CD27-	naive	12,627
**3**	CD38+ CD80high	ASC	2,574
**4**	IgD- CD27+	switched memory	27,931

Figure S9. PBMC sorting strategy to phenotype VRC01-engineered cells. A) Example gating strategy for PBMCs from animal 12093 14-days after prime showing how ASCs, ASC-like, naïve, and SM populations are identified. **B)** Four populations could be simultaneously sorted using the BD AriaFusion. A table showing sorting results from (A**)** is given. These cells were processed to detect VRC01 mRNA to identify the phenotypes of engineered cells after vaccination.

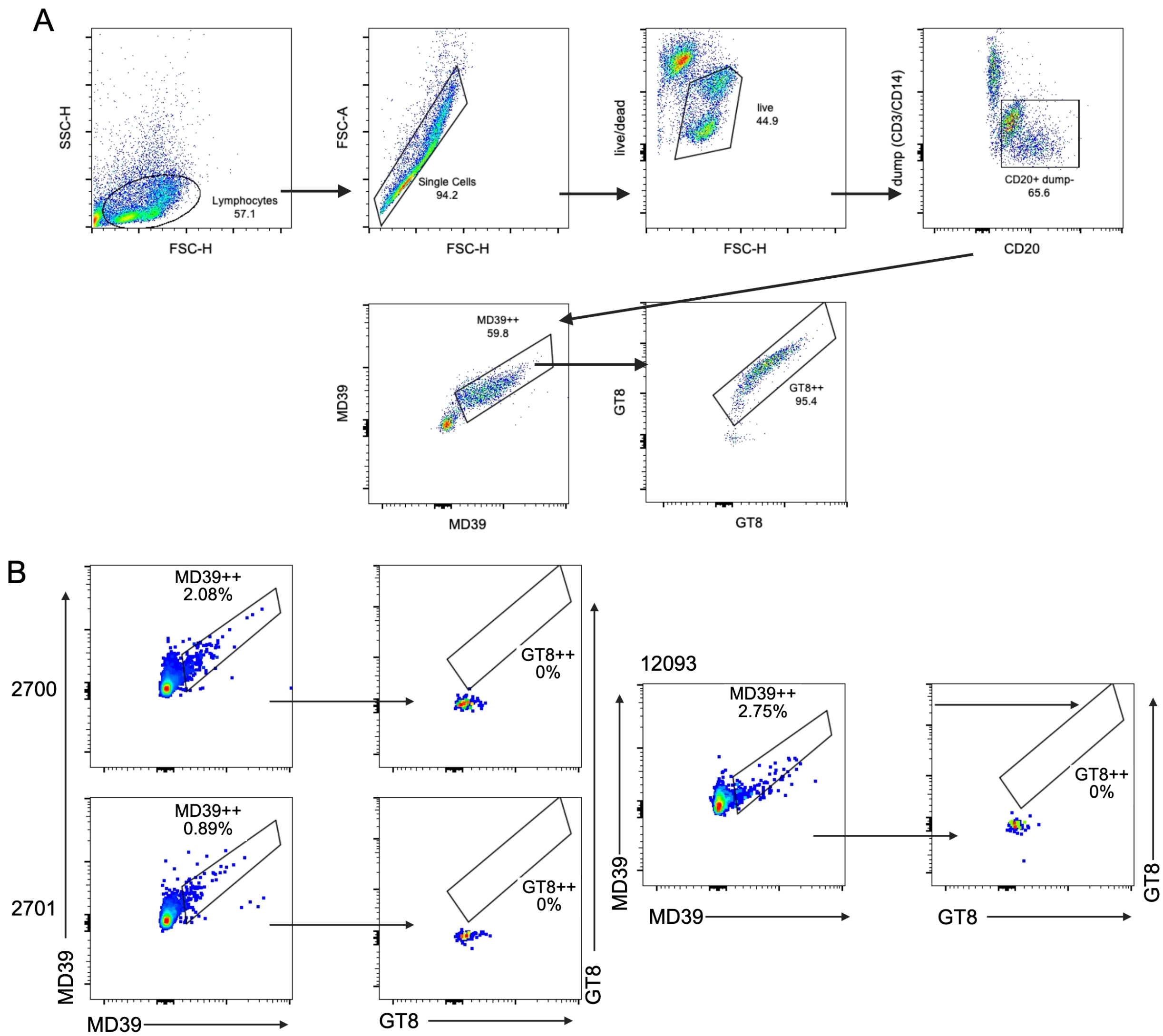

Figure S10. Responses to MD39-ferritin vaccination in lymph nodes. A) Gating strategy to identify VRC01 B cells as exemplified using a mouse pro-B cell line that is expressing the VRC01 BCR. VRC01 expressing cells are uniquely capable of recognizing the CD4 binding site (GT8) epitope in the context of native MD39 trimer and can thus be identified as $MD39^{++}$/eOD-$GT8^{++}$. Endogenous responses to the MD39-native trimer in NHPs are expected to be incapable of also binding to eOD-GT8. **B)** $MD39^{++}$ B cells from the draining LNs of all pilot-1 animals do not also recognize GT8, suggesting this population does not contain engrafted VRC01-reprogrammed cells.

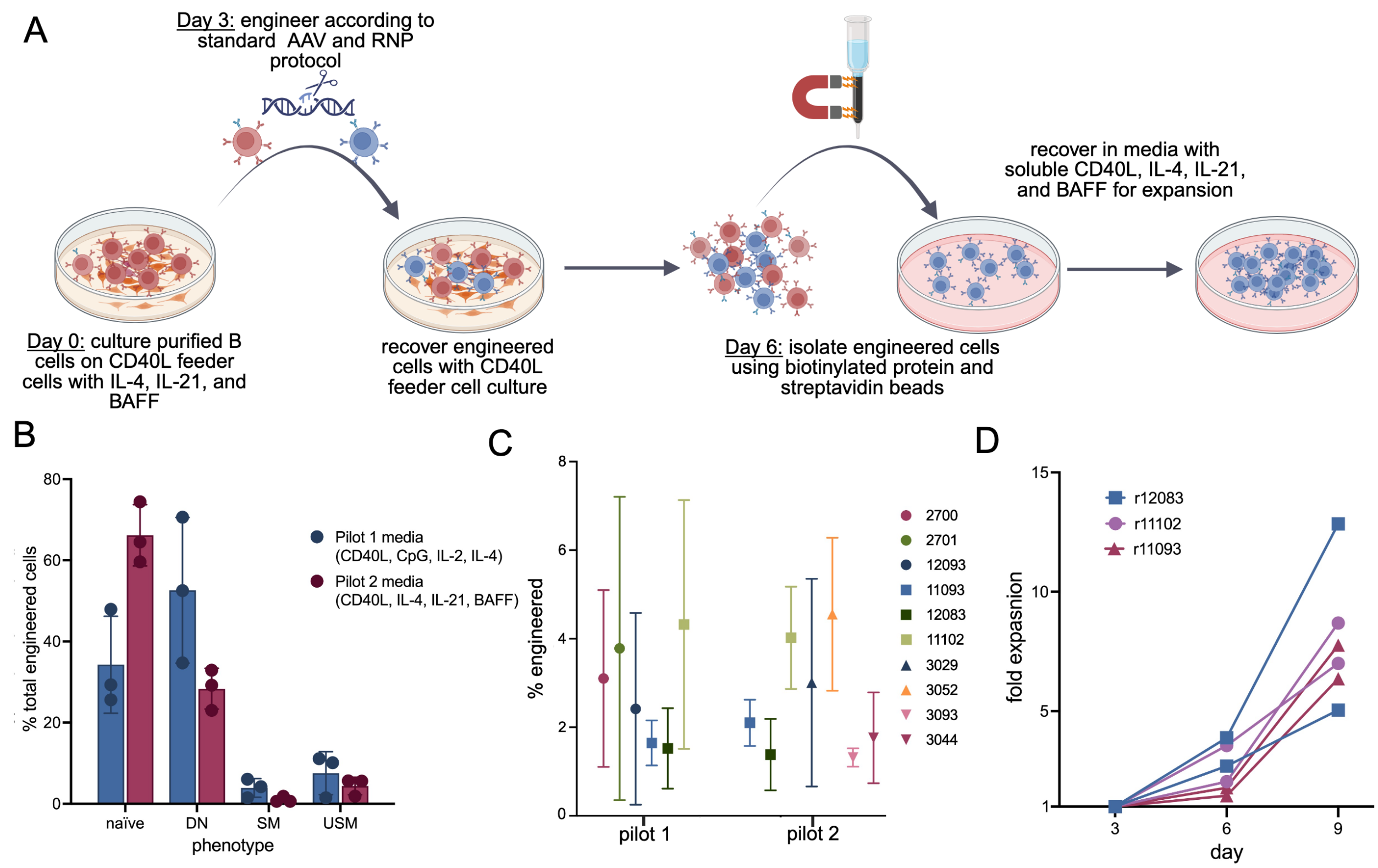

Figure S11. NHP B cell culture can be modified to expand *IgH*-reprogrammed cells and alter phenotype. A) Schematic of pilot-2 activation, engineering, expansion and MACS isolation. Cells are cultured with CD40L feeder cells, IL-4, IL-21, and BAFF beginning on day 0. On day 3, cells are engineered and recovered with the same CD40L feeder cell media. On day 6, engineered cells are isolated by MACS using biotinylated probes and streptavidin beads. These isolated engineered cells are cultured for three days with soluble CD40L instead of CD40L feeder cells. **B)** The frequency of phenotypes of VRC01 expressing $CD20^{+}$ B cells gated on live, single cells that are $KO11^{-}$ and $GT8^{++}$ in pilot-1 media (CD40L, IL-2, IL-4, CpG) compared to pilot-2 media (CD40L, IL-4, IL-21, and BAFF) at the time of engraftment. Data points are from experiments in different donor B cells. Error bars represent standard deviation from the mean. **C)** Comparison of VRC01 engineering efficiencies using pilot-1 and pilot-2 activation media across several donors. Engineering was performed the same way using both activation protocols, and efficiencies determined by flow cytometry three days after engineering. Data is given as mean from 2-6 experiments and error bars represent standard deviation from the mean **D)** Fold-expansion of VRC01 targeted cells for three donors (2 experiments each) when cultured in pilot-2 media, where expansion begins after engineering on day 3 of culture. Engineered cell population frequencies remained constant from day 6-9, as determined by flow cytometry.

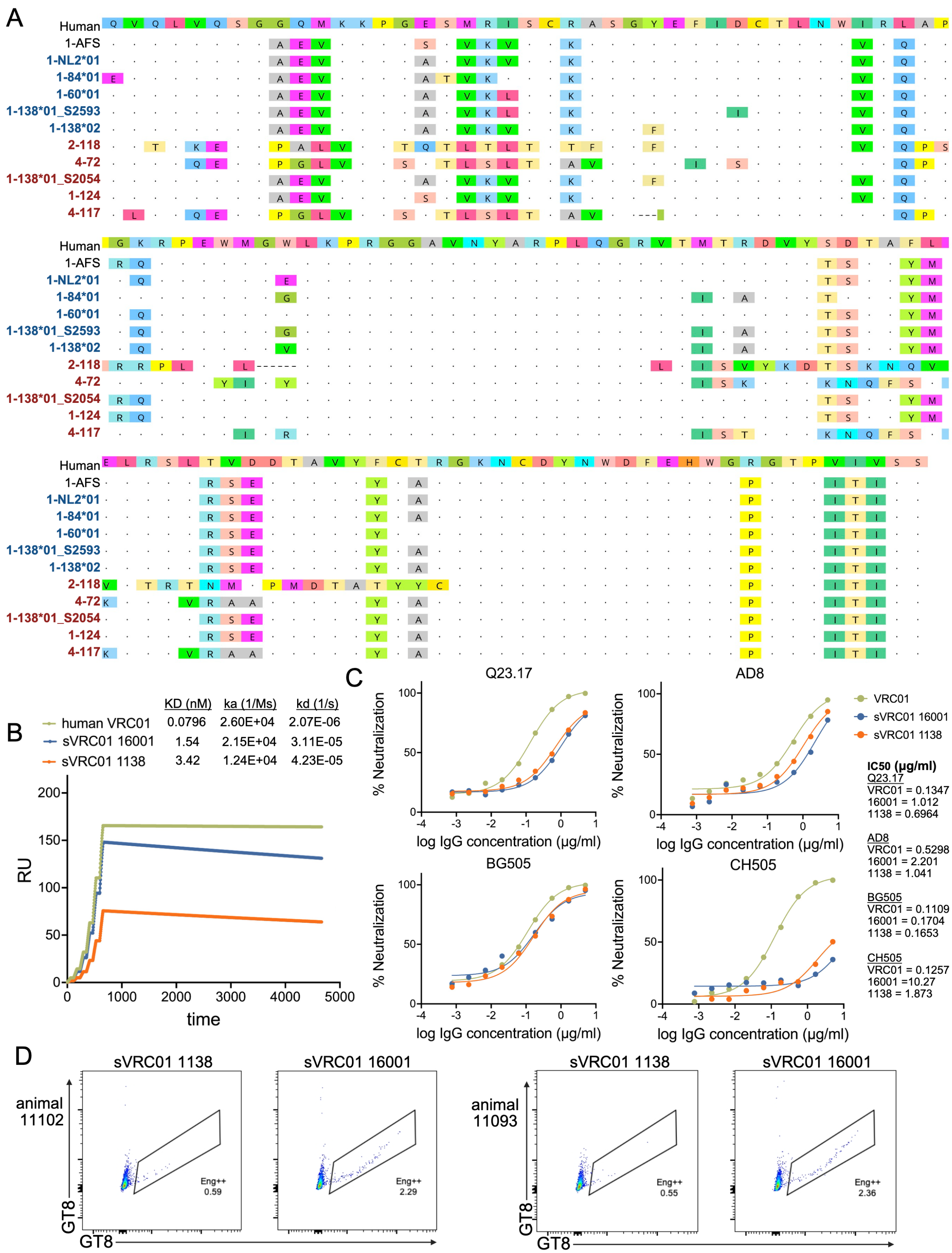

Figure S12. Simianization of VRC01. A) Alignment of sVRC01 compared to human VRC01 (VH only). Simianized versions of the VRC01 bnAb were generated by grafting CDRs and residues known to be important for antigen binding onto simian VH and VL genes, as previously described [79]. We designed 10

versions of sVRC01; 5 based on VH gene similarity to human VRC01 (red), and 5 based on frequency of VH genes in the macaca mulatta repertoire (blue). A single, previously published sVRC01 KLC was used in combination with these HCs. Of the 10 designs, only 1-138*01_S2054 and 1-60*01 expressed well as IgG (referred to as 1138 and 16001). **B)** Bivalent KDs of sVRC01 and human VRC01 with MD39 trimer by SPR. **C)** Neutralization of HIV pseudoviruses by human and simianized VRC01 recombinant monoclonal antibodies are given as a function of the log of the antibody concentration in µg/ml. IC50 values are given. **D)** 1138 and 16001 were made into AAV6 donor DNA cassettes and could be used to engineer cells from two donors (albeit at lower efficiency compared to the human sequence) as measured by flow cytometry with GT8 and K011 probes.

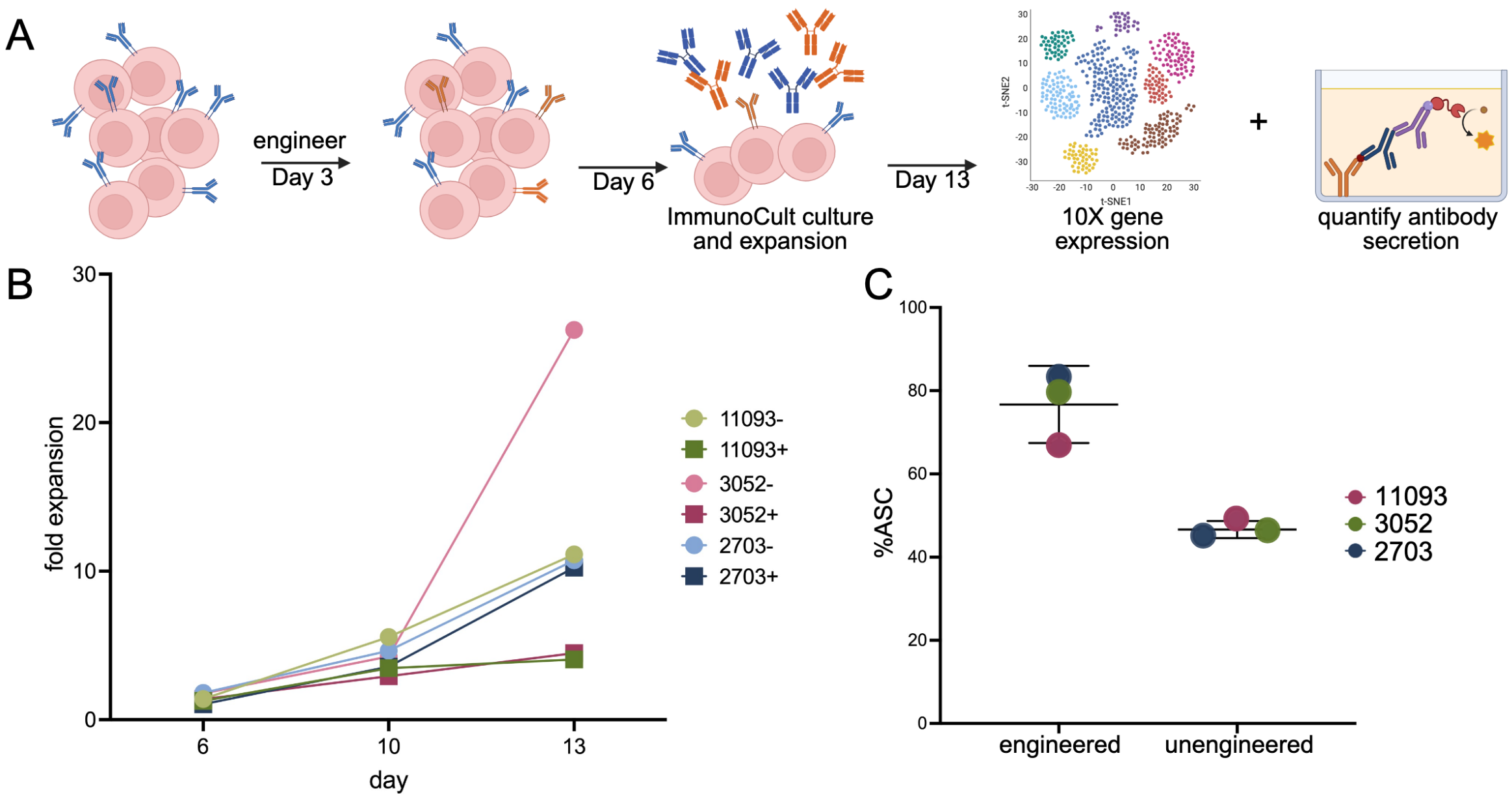

D

cluster	VRC01- total	VRC01- IgM	VRC01- IgG	VRC01- IgA	VRC01- IgE	VRC01+ total	VRC01+ IgM	VRC01+ IgG	VRC01+ IgA	VRC01+ IgE	% VRC01+
0	657	236	215	126	0	27	16	14	4	0	3.9
1	550	2	2	3	0	38	0	0	0	0	6.5
2	546	135	162	70	0	24	8	9	4	0	4.2
3	472	1	1	0	0	5	0	0	0	0	1.0
4	368	4	1	1	0	23	0	0	0	0	5.9
5	279	2	0	0	0	1	0	0	0	0	0.36
6	232	8	1	2	0	3	1	0	1	0	1.3
7	128	2	1	0	0	0	0	0	0	0	0.0

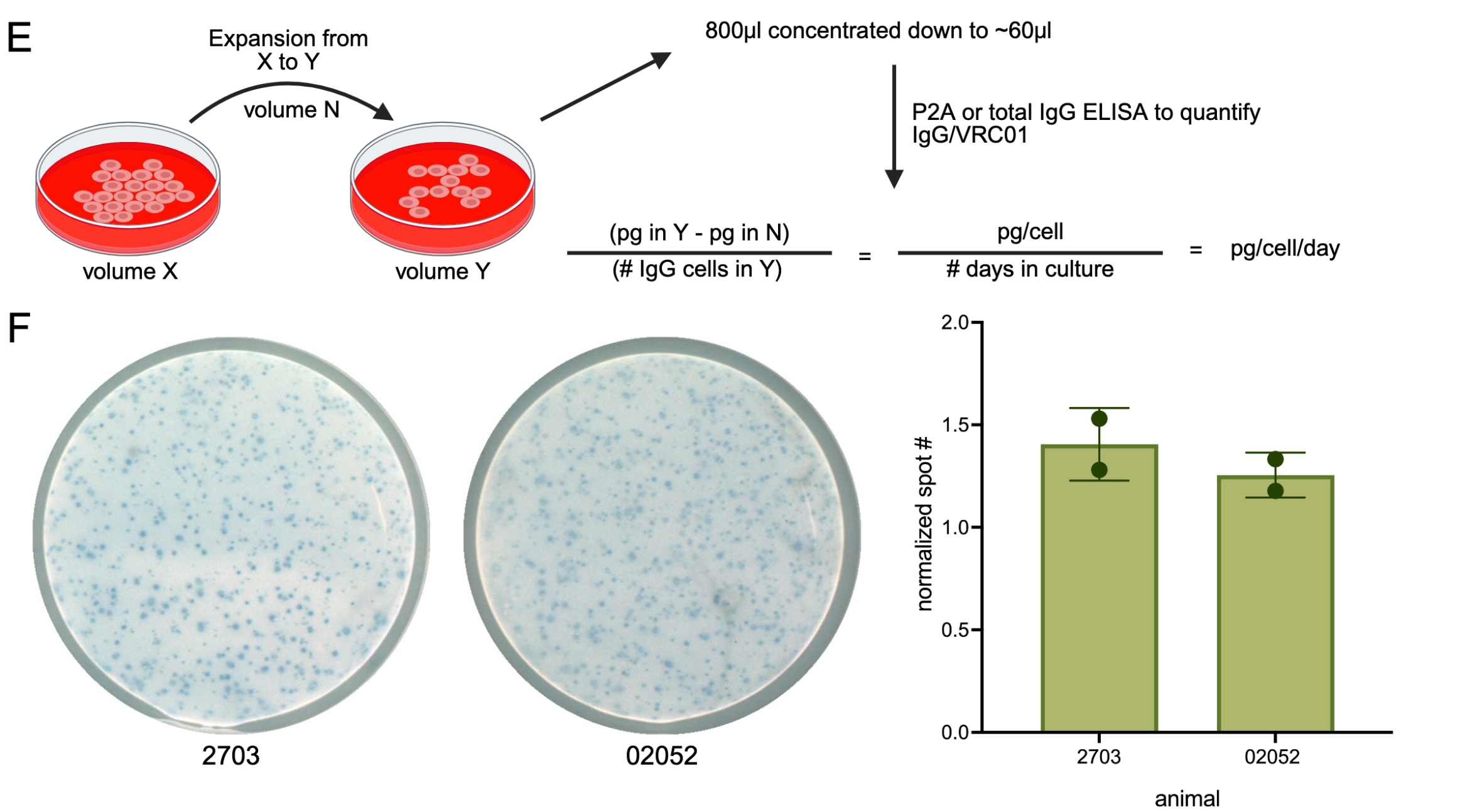

Figure S13. Extended ImmunoCult culture data. A) Schematic of ImmunoCult culture strategy and analysis. **B)** Fold expansion of ImmunoCult cultured engineered (+) and unengineered (-) cells from three donors, where the first expansion timepoint is determined on day 6, 3 days after engineering. **C)** Frequency of ASCs (CD38+CD80+) in engineered and unengineered cells in ImmunoCult cultures on day 13 of culture as determined by flow cytometry. **D)** Table of the total number of cells, as well as number of cells per

isotype, in each cluster for engineered (VRC01+) and unengineered (VRC01-) cells determined by 10X single cell transcriptomics on day 13 of ImmunoCult culture. Additionally, the percent of engineered cells in each cluster is shown. Isotype was determined based on raw expression values of *IgH* constant genes above an expression threshold of 250. **E)** Schematic of how VRC01 and total IgG was calculated in pg/cell/day. Cells were expanded in ImmunoCult media, where a portion of cells in media was removed every third day to expand and maintain cell density of 150k cells/ml in a 3ml culture. **F)** Results of VRC01 specific ELISpot, where plates were coated with anti-P2A antibody (specific for our VRC01 engineered cells), incubated with ImmunoCult engineered and expanded cells overnight, and spots detected using anti-human IgG. A total of ~4,000 engineered cells were added per well, with representative wells for both donors using the assay (left) and normalized spot counts (right) where normalized spot count = seeded # cells/resulting # spots.

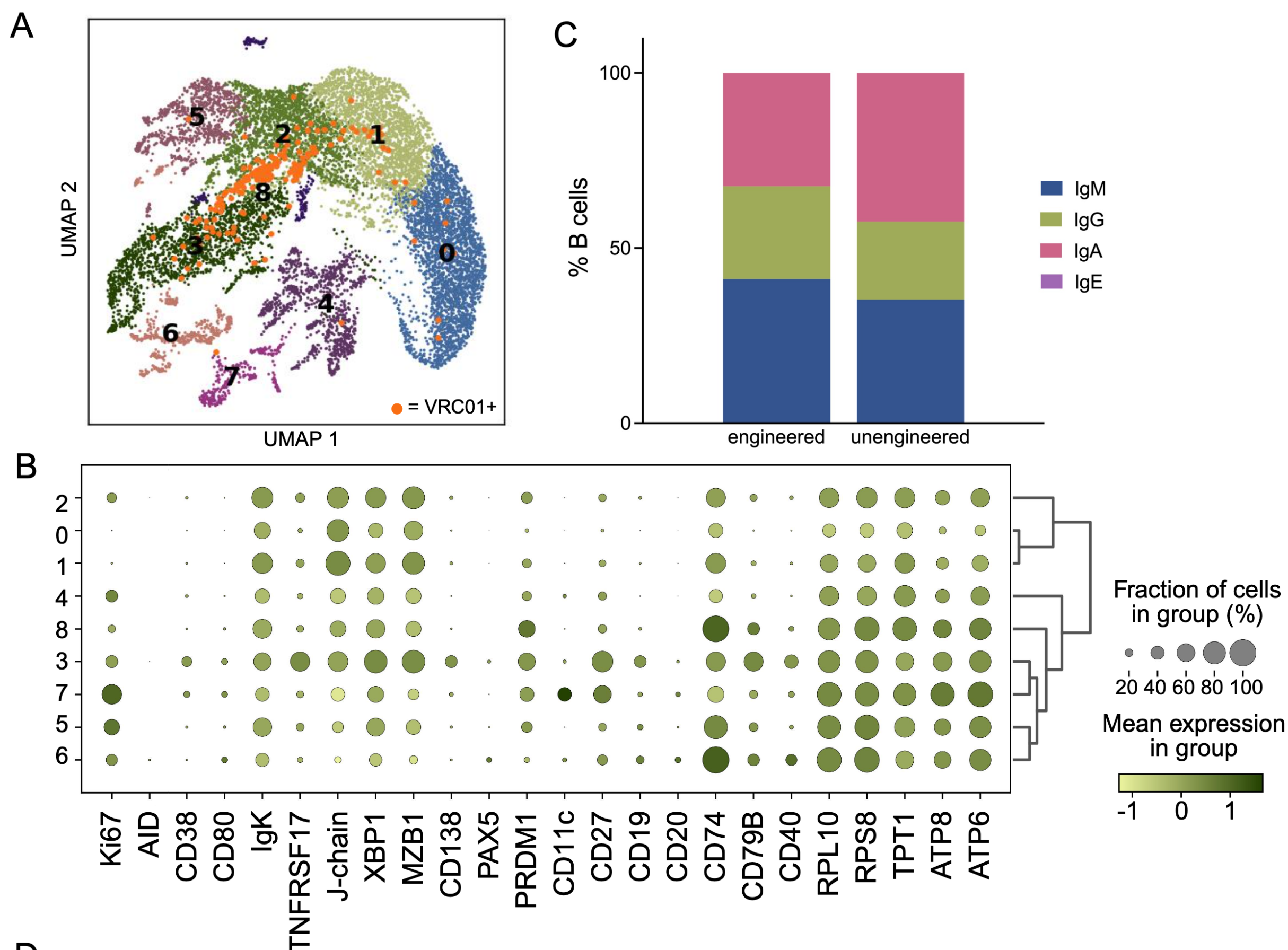

D

cluster	VRC01-					VRC01+					% VRC01+
	total	IgM	IgG	IgA	IgE	total	IgM	IgG	IgA	IgE	
0	2974	1	0	1	0	7	0	0	0	0	0.2
1	2463	12	15	10	0	51	1	0	0	0	2.0
2	2060	3	9	6	0	115	0	0	0	0	5.3
3	1826	431	258	532	0	37	13	8	10	0	2
4	1036	12	1	0	0	1	0	0	0	0	0.1
5	899	1	0	0	0	1	0	0	0	0	0.1
6	534	2	6	10	0	0	0	0	0	0	0
7	337	15	11	15	0	1	0	1	1	0	0.3
8	181	0	0	0	0	0	0	0	0	0	0

Figure S14. Characterization of *IgH*-reprogrammed B cells after *in vitro* differentiation by scRNAseq. A) UMAP Leiden clusters from scRNAseq data of an engineered pool of cells cultured to become ASCs *in vitro*. Cells were cultured, targeted, and expanded for 13 days in ImmunoCult B cell expansion media. Engineered cells maintained a frequency of ~2% for the duration of culture. **B)** Dotplot showing transcriptional levels of genes of interest for cells in **A**, and relatedness of each cluster is shown as a phylogenic tree. Engineered cells are enriched in clusters 0-3, which are defined by decreased expression of canonical B cell markers and increased expression of Ig transcripts and genes associated with ASCs, such as XBP1 and MZB1. Engineered cells are also present in cluster 8, which maintains some transcription of naive B cell markers as well as some transcription of ASC-associated genes, suggesting differentiation toward an ASC phenotype. This cluster, along with clusters 5 and 6, show high levels of CD74 transcription, which was observed in the other sample sequenced at the same timepoint (Fig. 6B). **C)** Comparison of isotype frequencies of engineered cells compared to unengineered cells. Isotype was determined based on raw expression values of *IgH* constant genes above an expression threshold of 250. Due to low sequencing depth, isotype was determined for a smaller fraction of cells, as compared to the other ImmunoCult expanded sample (Fig. 4C-E). **D)** Table of the total number of cells, as well as number of cells

per isotype, in each cluster for engineered (VRC01+) and unengineered (VRC01-) cells determined by 10X single cell transcriptomics on day-13 of ImmunoCult culture. Additionally, the percent of engineered cells in each cluster is shown.

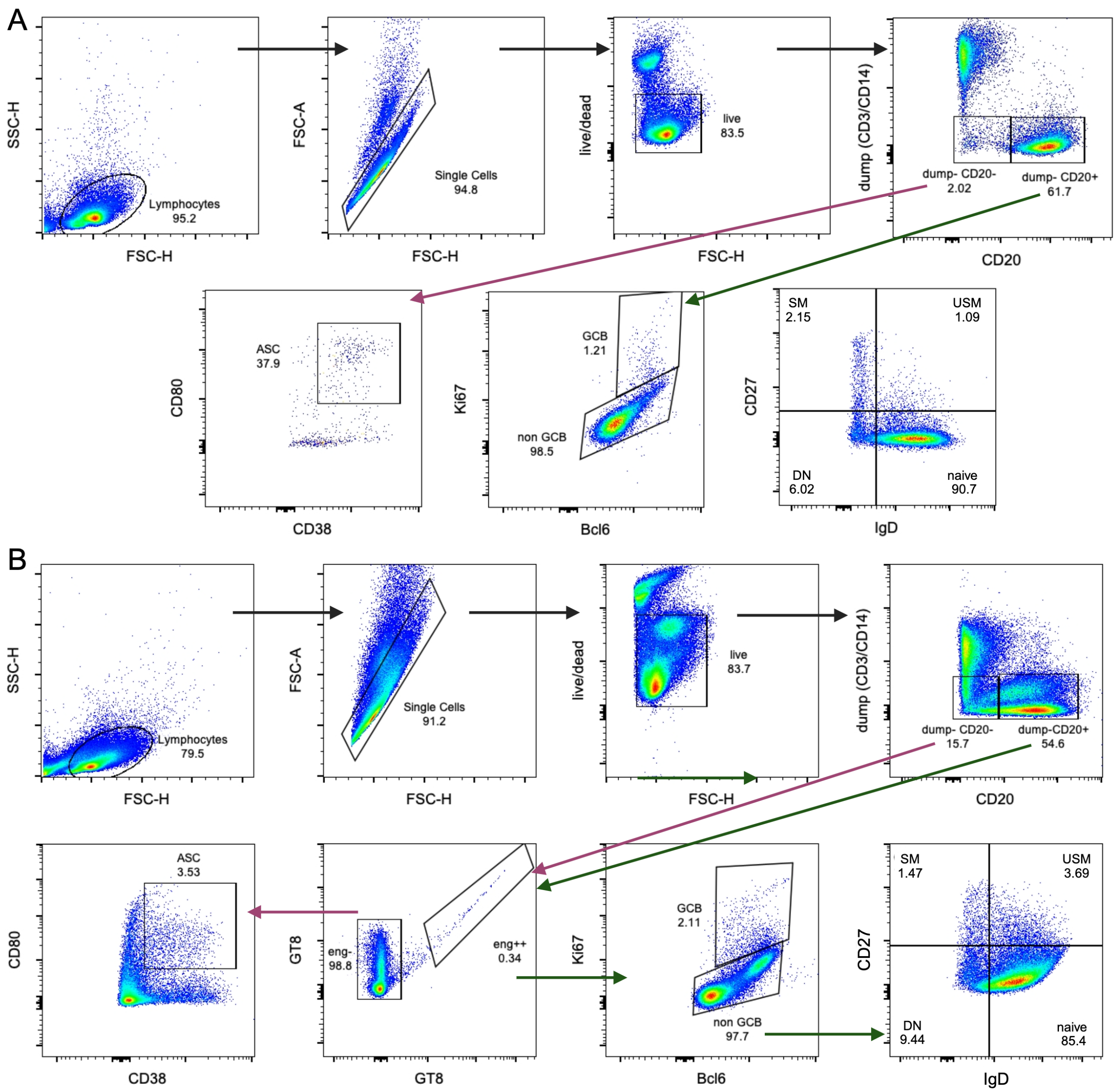

Figure S15. A) Example organoid B cell phenotype gating strategy where CD20+ B cells are gated as live, single lymphocytes. This population is then gated into GCBs or non-GCBs based on Ki67 and Bcl6 expression (green arrow). Non-GCBs are further gated as SM, USM, DN, or naïve based on CD27 and IgD expression (green arrows). ASCs are defined as CD38+CD80+ from the dump-CD20- population (pink arrow). **B)** Example organoid gating strategy as in (A**)** with engineered cells added. An intracellular antigen staining step is used to identify engineered ASCs.

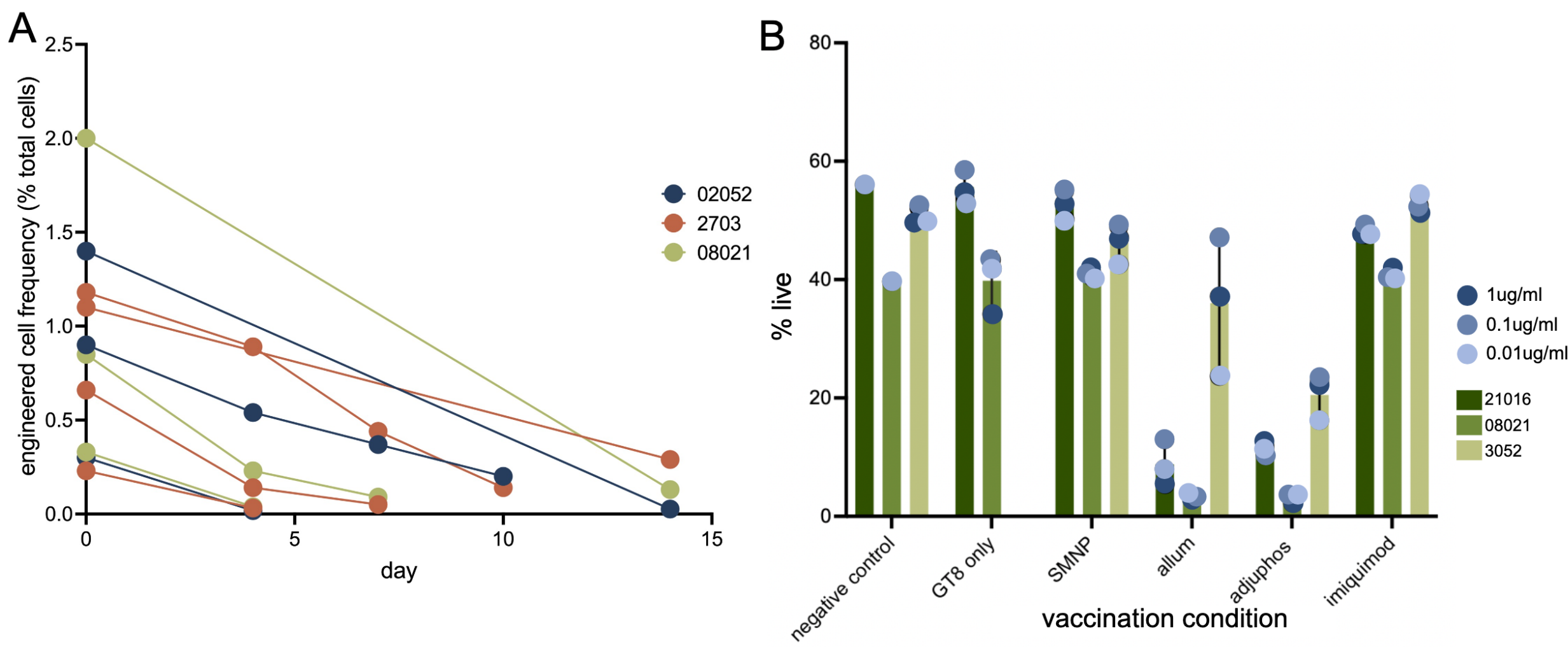

Figure S16. Organoids derived from SLO tissue can be used to evaluate engineered cell responses to HIV Env based immunogens. A) Frequency of engineered cells in organoid cultures over time across multiple experiments for three donors without vaccination. **B)** A titration of immunogen and a variety of adjuvants were tested for compatibility with our organoid culture system. Here, we show the viability of eOD-GT8-60mer vaccinated organoids on day-14 of culture across 3 donor animals. Vaccinations were performed on day-0.
